# Supplementary material for: Immunogenicity and safety of Quadrivalent Influenza HA vaccine compared with Trivalent Influenza HA vaccine and evaluation of Quadrivalent Influenza HA vaccine batch-to-batch consistency in Indonesian children and adults
Source: PLoS One. 2023 Aug 24;18(8):e0281566. doi: 10.1371/journal.pone.0281566 (PMC10449119; doi:10.1371/journal.pone.0281566)
Supplement: S1 File — (PDF) [file pone.0281566.s002.pdf]

Protectivity and Safety of Quadrivalent Influenza HA Vaccine in Indonesian Population
(Bridging Study)

QIV 0217

CLINICAL TRIAL PROTOCOL

Sponsor **PT BIO FARMA (PERSERO)**
Jl. Pasteur No. 28 Bandung – 40161 Indonesia

August 2017

Protectivity and Safety of Quadrivalent Influenza HA Vaccine in Indonesian Population (Bridging Study)

QIV 0217

STUDY PROTOCOL

Sponsor	P.T.BIO FARMA (PERSERO)<br>Jl.Pasteur No.28 Bandung – 40161 INDONESIA
Investigational Product	Quadrivalent Influenza Vaccine (Bio Farma)
Manufacturing Sites	PT Bio Farma, Jl.Pasteur no. 28 Bandung – 40161 Indonesia
Principal Investigator	Dr. Meita Dhamayanti, dr.,Sp.A(K).,MKes.
Medical Advisor	Prof. Cissy B. Kartasasmita, dr.,Sp.A(K).,MSc.,PhD.
Subinvestigators	1. Prof. Dr. Kusnandi Rusmil, dr.,Sp.A(K).,MM.<br>2. Dr. Eddy Fadlyana, dr.,Sp.A(K).,MKes.<br>3. Rodman Tarigan, dr.,Sp.A(K).,Mkes.<br>4. Susantina Prodjosoewojo, dr.,Sp.PD<br>5. Andri Reza Rahmadi, dr.,Sp.PD.,MKes.
Biometry	Dr. Drs. Hadyana Sukandar, MSc.
Biological Laboratory	Rini Mulia Sari, dr.<br>Yani Sukriyani<br>Restika Andiarini
Monitor	Dr. Novilia Sjafri Bachtiar, dr., M.Kes.<br>Rini Mulia Sari, dr<br>Julianita Fahmi, dr<br>Asep Irham F. Q., dr.
Date	2017
Version	1.

TABLE OF CONTENTS

SYNOPSIS 6
FLOW CHART.. 10
Schedule & Activities 11
Abbreviations and Symbols Used 12
Glossary 13
1. Background and Rationale 15
1.1. Introduction 15
1.2. Epidemiology 16
1.3. Prevention and Control of Infection among Humans 18
1.4. Rationale 18
1.5 Previous Study 19
2. Objectives.. 23
2.1. Primary objective 23
2.2. Secondary Objectives.. 23
3. Trial Design and Methodology 24
3.1. Trial Design 24
3.2. Treatment Allocation Procedures.. 25
3.3. Trial Population.. 26
3.3.1. Inclusion Criteria 26
3.3.2. Exclusion Criteria 26
3.3.3. Prior and Concomitant Therapy 26
3.4. Trial Plan.. 27
3.4.1. Conditions for conducting the trial 27
3.4.2. Trial Calendar/Timelines.. 29
3.4.3. Trial Center 29
3.4.4. Vaccination and Serology Schedule.. 29
3.4.5. Procedure for Obtaining, Handling and Shipment of Serum Samples...... 29
3.5. Case Report Form and Data Collection 31
3.6. Subject Diaries and Interim Histories 31
3.7. Clinical Supplies 32
4. Products.. 33
4.1. Investigational Product Characteristics: Quadrivalent Influenza HA vaccine.33
4.1.1. Product Description.. 33

4.1.2. Composition ... 33
4.2 Control Product Characteristics: Trivalent Influenza HA vaccine ... 33
4.2.1. Product Description ... 34
4.2.2. Composition ... 34
4.3. Preparation ... 34
4.3.1. Precautions for use ... 34
4.4. Labeling and Packaging ... 35
4.5. Storage and Shipment Conditions ... 35
4.5.1. Shipment Conditions ... 35
4.5.2. Storage Conditions ... 35
4.6. Accountability ... 35
4.7. Return of Unused Products ... 36
5. Trial Administration ... 37
5.1. Personnel involved in the trial ... 37
5.2. Visit Procedures ... 37
5.3. Conditions for Withdrawal from the Trial ... 39
5.4. Lost to Follow-up Procedures ... 39
5.5. Termination Classification ... 39
5.6. Monitoring, Auditing and Archiving ... 40
5.6.1. Routine Monitoring ... 40
5.6.2. Audits and Inspections ... 41
5.6.3. Archiving ... 41
6. Adverse Event Reporting ... 42
6.1. Definitions ... 42
6.2. Expected Reactions ... 43
6.3. Safety Data Collection and Management Procedures ... 43
6.4. Reporting of Serious Adverse Events ... 44
6.5 Causality Assessment ... 45
7. Evaluation Criteria ... 49
7.1. Primary Evaluation Criteria ... 49
7.1.1. Definition of the Criteria ... 49
7.1.2. Parameters to be measured ... 49
7.1.3. Method and Timing of Measurement ... 49
7.2. Secondary Evaluation Criteria ... 49
7.2.1. Definition of the Criteria ... 49

7.2.2. Parameters to be measured..50
8. Biometry 52
8.1. Statistical Methods and Data Analysis..52
8.1.1. Determination of Sample Size 52
8.1.2. Data sets to be analyzed...52
8.1.3. Statistical Methodology 53
8.2. Data Management 54
9. Confidentiality, Stipends and Adverse Events Compensation and Insurance..........55
9.1. Confidentiality...55
9.1.1. Confidentiality of Data...55
9.1.2. Confidentiality of Patient Records 55
9.2. Stipends for Participation 55
9.3. Adverse Events Compensation and Insurance 55
10. Publication Policy 56
11. Bibliographical References 57
12. Appendices...60
Appendix 1: Personnel Involved in the Trial 61
Appendix 2: Sample SAE reporting forms 62
Appendix 3: Plastic bangle model 64

SYNOPSIS

**Sponsor**	PT. Bio Farma (Persero)
**Trial Title**	Protectivity and Safety of Quadrivalent Influenza Vaccine in Indonesian Population (Bridging Study)
**Investigational Product**	Quadrivalent Influenza Vaccine (Bio Farma)<br>**Composition (0.5 ml):**<br>60 µg haemagglutinin Quadrivalent Influenza<br>4 µg thimerosal
**Manufacturing Site**	PT. Bio Farma (Persero)<br>Jl. Pasteur No. 28 Bandung, Indonesia
**Principal Investigator**	Dr. Meita Dhamayanti, dr.,Sp.A(K).,MKes.
**Sub-Investigators**	1. Prof. Dr. Kusnandi Rusmil, dr.,Sp.A(K).,MKes.<br>2. Dr. Eddy Fadlyana, dr.,Sp.A(K).,MKes.<br>3. Rodman Tarigan, dr.,Sp.A(K).,Mkes.<br>4. Susantina Prodjosoewojo, dr.,Sp.PD<br>5. Andri Reza Rahmadi, dr.,Sp.PD.,MKes.
**Medical Advisor**	Prof. Cissy B. Kartasasmita, dr.,Sp.A(K).,MSc.,PhD.
**Biometry**	Dr. Drs. Hadyana Sukandar, MSc.
**Monitor**	Dr. Novilia Sjafri Bachtiar, dr., M.Kes.
	Rini Mulia Sari, dr.
	Julianita Fahmi, dr
	Asep Irham F. Q., dr
**Biological Laboratory**	Rini Mulia Sari, dr.
	Yani Sukriyani
	Restika Andiarini
**Trial Centre**	Hasan Sadikin General Hospital/Medicine Faculty-Padjadjaran University, Bandung
**Trial Phase**	Bridging study
**Protocol Number**	QIV 0217
**Study Period**	12 months
**Primary Objective**	To assess the protectivity rate of Quadrivalent Influenza HA vaccine 28 days after immunization in Indonesian population
**Secondary Objectives**	o To describe immunogenicity of quadrivalent Influenza HA vaccine in all subjects<br>o To assess the safety of quadrivalent Influenza HA vaccine in all subjects<br>o To evaluate immunogenicity and safety one dose of quadrivalent influenza HA vaccine compare to trivalent Influenza HA vaccine in group 9-40 years of age<br>o To evaluate immunogenicity and safety in three consecutive batches of quadrivalent Influenza HA vaccine in group 9-40 years of age.

**Trial Design**	**Subjects 9- 40 years of age**<br>Experimental, randomized, double blind bridging study, four - arm parallel group study in an estimated 540 subjects (135 subjects per arm)<br><br>**Subjects 6 months - 8 years of age:**<br>Experimental, open labeled bridging study, two-arm parallel group study in an estimated 270 subjects (135 subjects per arm)
**Planned Sample Size**	- 540 subjects (9-40 years of age)<br>- 270 subjects (6 months-8 years of age)
**Inclusion Criteria**	**Subjects 9- 40 years of age**<br>1. Healthy<br>2. Properly informed about the study and having signed the informed consent form<br>3. Subject/Parent will commit themselves to comply with the instructions of the investigator and the schedule of the trial.
	**Subjects 6 months - 8 years of age:**<br>1. Healthy<br>2. Parents have been informed properly regarding the study and signed the informed consent form<br>3. Parents will commit themselves to comply with the instructions of the investigator and the schedule of the trial
**Exclusion Criteria**	1. Subject concomitantly enrolled or scheduled to be enrolled in another trial<br>2. Evolving mild, moderate or severe illness, especially infectious diseases or fever (axillary temperature ≥ 37.5°C)<br>3. Known history of allergy to egg and or chicken protein or any component of the vaccines<br>4. History of uncontrolled coagulopathy or blood disorders contraindicating intramuscular injection<br>5. Subject who has received in the previous 4 weeks a treatment likely to alter the immune response (intravenous immunoglobulins, blood-derived products or long term corticotherapy (> 2 weeks).<br>6. Pregnancy & Lactation (Adult)<br>7. Any abnormality or chronic disease which according to the investigator might interfere with the assessment of the trial objectives<br>8. Subject already immunized with influenza vaccine within 1 year.<br>9. Subjects receive any vaccination within 1 month before and after immunization of Quadrivalent Influenza Vaccine.

<table>
<tr><td rowspan="7">Test Product</td><td>Investigational Product</td><td>Quadrivalent Influenza HA Vaccine</td></tr>
<tr><td>Form</td><td>Liquid in vial</td></tr>
<tr><td>Dose</td><td>0.5 ml (Each dose of vaccine (0.5ml) Quadrivalent Influenza Vaccine composed of: 15 µg HA of each strain*, thimerosal 4 µg)</td></tr>
<tr><td>Batch Number</td><td>A: 3070117
B: 3070217
C: 3070317</td></tr>
<tr><td colspan="2">List of Strains:
A/California/7/2009 (X-179A)(H1N1)pdm09
A/Hong Kong/4801/2014(X-263) (H3N2)
B/Texas/2/2013
B/Phuket/3073/2013</td></tr>
<tr><td colspan="2">Subjects 9- 40 years of age
<table>
<tr><th>Group</th><th>Age</th><th>Number of Dose</th><th>Vaccine Dose</th></tr>
<tr><td>I</td><td>9 - 12 years</td><td>1</td><td>0.5 ml QIV/TIV</td></tr>
<tr><td>II</td><td>13-17 years</td><td>1</td><td>0.5 ml QIV/TIV</td></tr>
<tr><td>III</td><td>18- 40 years</td><td>1</td><td>0.5 ml QIV/TIV</td></tr>
</table>
Each group will receive quadrivalent influenza vaccine with different batch number (batch 1/ batch 2 / batch 3) or trivalent influenza vaccine as a control.</td></tr>
<tr><td colspan="2">Subjects 6 months - 8 years of age
<table>
<tr><th>Group</th><th>Age</th><th>Number of Dose</th><th>Vaccine Dose</th></tr>
<tr><td>IV</td><td>6 – 35 months</td><td>2</td><td>0.25ml</td></tr>
<tr><td>V</td><td>3-8 years</td><td>2</td><td>0.5ml</td></tr>
</table>
Each group will receive quadrivalent influenza vaccine with batch number: 3070217</td></tr>
<tr><td>Vaccination Schedule</td><td colspan="2">Subjects 9- 40 years of age
Immunization schedule:
V1 (Day 0) : 1st dose of Influenza HA Vaccine

Subjects 6 – 35 months of age and 3-8 years of age:
V1 (Day 0) : 1st dose of Influenza HA vaccine
V2 (Day 0 + 28 (-4/+7)days): 2nd dose of Influenza HA vaccine</td></tr>
<tr><td>Serology Schedule</td><td colspan="2">Subjects 9- 40 years of age
V1 (Day 0) : before immunization
V2 (Day 0 + 28 (-4/+7)) days

Subjects 6 months - 8 years of age
V1 (Day 0) : before immunization</td></tr>
</table>

	V3 (Day 0 + 56 (-4/+7)) days
**Evaluation Criteria**	
**Primary Evaluation Critera**	Percentage of subjects with anti-HI titer ≥ 1:40, 28 days after Influenza HA vaccination.
**Secondary Evaluation Criteria**	**Immunogenicity**<br>o Serological response to Influenza HA vaccine: GMT, percentage of subjects with increasing antibody titer ≥ 4 times and/or percentage of subjects with transition of seronegative to seropositive following vaccination.<br>o Description of serological response between one dose of quadrivalent and trivalent influenza HA vaccine in subjects 9-40 years old<br>o Description of serological response between each batch number of Quadrivalent Influenza HA vaccine in subjects 9-40 years old<br><br>**Safety**<br>o Immediate reactions within the first 30 minutes after each injection<br>o Local and systemic events occurring within 72 h after each injection<br>o Local and systemic events occurring between 72 h and 28 days following injection.<br>o Any serious adverse event occurring from inclusion until 28 days after immunization<br>o Description of adverse events between quadrivalent and trivalent influenza HA vaccine.<br>o Description of adverse events between each batch number

FLOW CHART

A. Subjects 9- 40 years of age

Group	Age	Vaccine				Vaccine dose
		QIV Batch 1	QIV Batch 2	QIV Batch 3	TIV	
I	9 – 12 years	45	45	45	45	0.5 ml
II	13– 17 years	45	45	45	45	0.5 ml
III	18 – 40 years	45	45	45	45	0.5 ml
Total Subjects		135	135	135	135	-

Each group will receive 1 dose of quadrivalent influenza vaccine with different batch number (batch 1/ batch 2 / batch 3) or trivalent influenza vaccine as a control.

B. Subjects 6 months - 8 years of age

Group	Age	Number of Dose	Vaccine Dose
IV	6 – 35 months	2	0.25ml
V	3-8 years	2	0.5ml

Each group will receive 2 doses of quadrivalent influenza vaccine from 1 batch number.

Study Scheme

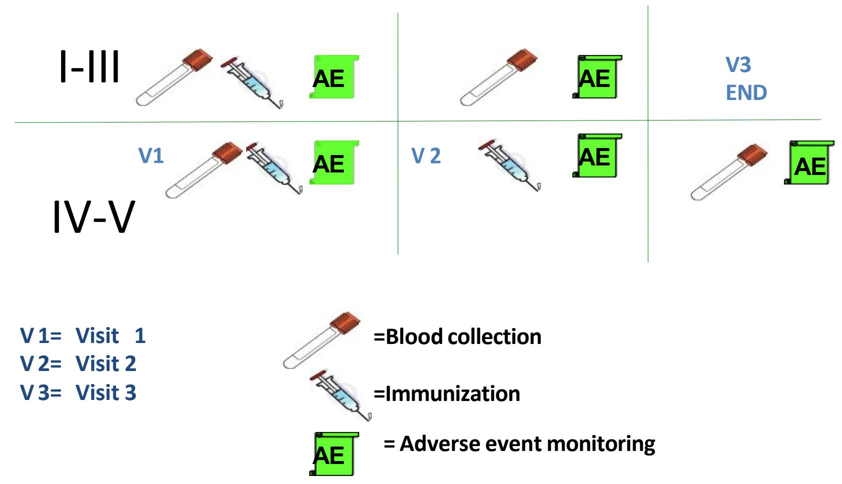

Visit Number	V1	V2	V3
Visit Intervals (Days)	Day 0	V1 +28	V2+28D<br>(IV & V)
Time Windows (Days)	-	(-4/+7)D	(-4/+7)D
Informed consent signed	X		
Inclusion & Exclusion Criteria	X		
Past Medical History	X		
Physical Examination	X	X	X
Blood Sampling	X	X<br>I-III	X<br>(IV & V)
Temporary and Definite Contraindications Review	X	X<br>(IV & V)	
Vaccination Dose	X<br>VAX1	X<br>(IV & V)	-
Immediate surveillance (30 min)	X	X<br>(IV & V)	-
Assessment of Local Reactions<br>& Systemic Events	X	X	X<br>(IV & V)
Diary Cards Provided	X	X<br>(IV & V)	-
Diary Cards Collected		X	X
Prior & Concomitant Therapies	X	X	X
Termination Record/Final		X	X
Adverse Events/Serious Adverse Events	X	X	X

Abbreviations and Symbols Used

ACIP	Advisory Committee on Immunization Practices
AAP	AmericanAcademy of Pediatrics
95% CI	95% Confidence Interval
CRF	Case Report Form
D	Day (D1=Day1)
DC	Diary Card
GCP	Good Clinical Practice
GMT	Geometric Mean Titer
HA	Haemagglutination
HI	Haemagglutination Inhibition
IEC	Institutional Ethics Committee
IRAEF	Initial Report for Adverse Event Form
IRB	Institutional Review Board
QIV	Quadrivalent Influenza HA Vaccine
SAE	Serious Adverse Event
TIV	Trivalent Influenza HA Vaccine
V	Visit (V1=Visit1)
VAX	Vaccination
WHO	World Health Organization

Glossary

1. **Blinding**; a procedure in which one or more parties to the trial are kept unware of the treatment assignment(s). In case of measurement blinding, the technician being unware of the measure sample(s) before or after immunization.
2. **Bridging Study;** a study performed in the new region to provide pharmacodinamic or clinical data on efficacy, safety, dosage and dose regimen in the new region.
3. **Case report form (CRF)**; A printed, optical, or electronic document designed to record all of the protocol required information to be reported to the sponsor on each trial subject.
4. **Clinical Trial/Study;** Any investigation in human subjects intended to discover or verivy the clinical, pharmacological and/or other pharmacodynamic effects of an investigational product(s), and/or identify any adverse reactions to an investigational product(s), and/or to study absorption, distribution, metabolism, and excretion of an investigational product(s) with the objects of ascertaining its safety and/or efficacy. The terms clinical trial and clinical study synonymous.
5. **Impartial witness;** a person, who is independent of the trial, who cannot be unfairly influenced by people involved with the trial, who attend the informed consent process if the subject or the subject's legally acceptable representative cannot read, and who reads the informed consent form and any other written information supplied to the subject.
6. **Immunogenicity;** capabality of inducing an immune reponse.
7. **Informed consent;** a process by which a subject voluntary confirms his or her willingness to participate in a particular trial, after having been informed of all aspects of the trial that are relevant to the subject's decision to parcipate. Informed consent in documented by means of a written, signed and dated informed consent form.
8. **Investigator;** a person responsiblefor the conduct of the clinical trial at a trial site. If a trial is conducted by a team of individuals at a trial site, the investigator is the responsible leader of the team and may be called the principal investigator. See also subinvestigator.
9. **Monitoring;** the act of overseeing the progress of a clinical trial, and of ensuring that it is conducted, recorded, and reported in accordance with the protocol, SOPs (Standard Operating procedures), GCP (Good Clinical Practice) and the applicable regulatory requirement(s).
10. **Sponsor;** an individual, company, institution, or organization which takes responsibility for the initiation, management, and/or financing of a clinical trial.

11. **Subinvestigator;** any individual member of the clinical trial team designated and supervised by the investigator at a trial site to perform critical trial-related procedures and/or to make important trial-related decisions (e.g., associates, residents, research fellows). See also investigator.

1. Background and Rationale

1.1. Introduction

The Global Action Plan for Influenza Vaccines (GAP) was launched by the World Health Organization (WHO) in 2006 to reduce the global shortage of seasonal and pandemic influenza vaccines. A major goal of GAP is to increase seasonal influenza vaccine use globally. Addressing this goal would prevent severe influenza illness, strengthen health systems within countries to better respond to influenza pandemics, and encourage the vaccine industry to develop greater influenza vaccine manufacturing capacity.[1, 2]

Seasonal influenza viruses circulate and cause disease in humans every year. Seasonal influenza viruses can cause mild to severe illness and even death, particularly in some high-risk individuals. Persons at increased risk for severe disease include pregnant women, the very young and very old, immune-compromised people, and people with chronic underlying medical conditions. Seasonal influenza viruses evolve continuously, which means that people can get infected multiple times throughout their lives. Therefore the components of seasonal influenza vaccines are reviewed frequently (currently biannually) and updated periodically to ensure continued effectiveness of the vaccines.[3] Recommendations are made in September for the following influenza season in the southern hemisphere and in February for the following influenza season in the northern hemisphere because approximately 6-8 months are needed to produce and approve vaccines. For countries in equatorial regions, epidemiological considerations influence which recommendation (February or September) individual national and regional authorities consider appropriate.[4]

---

[1]World Health Organization. Global action plan for influenza vaccines. Geneva: World Health Organization; 2016.
[2] Ortiz JR, Perut M, Dumolard L, et al.A global review of national influenza immunization policies: Analysis of the 2014 WHO/UNICEF Joint Reporting Form on immunization. Vaccine. 2016; 34:5400-05. http://dx.doi.org/10.1016/j.vaccine.2016.07.045
[3] World Health Organization. Influenza virus infections in humans (February 2014). Geneva: World Health Organization; 2014.
[4] World Health Organization. Questions and Answers Recommended composition of influenza virus vaccines for use in the northern hemisphere 2016-2017 influenza season and development of candidate vaccine viruses for pandemic preparedness. Geneva: World Health Organization; 2016.

According to the Laboratory Network in ILI and SARI Surveillance System for Influenza Virus Evolution Monitoring, the patterns of Influenza strain in Indonesia were compatible with global pattern of Northern Hemisphere. Thus, Northern Hemisphere Influenza strain was used in Indonesia.[5]

1.2. Epidemiology

Influenza is a highly infectious viral illness. In 1936, Burnet discovered that influenza virus could be grown in embryonated hens'eggs. This led to the study of the characteristics of the virus and the development of inactivated vaccines. The protective efficacy of these inactivated vaccines was determined in the 1950s. The first live attenuated influenza vaccine was licensed in 2003.[6]

There are three large groupings or types of seasonal influenza viruses, labelled A, B, and C. Type C influenza causes milder infections and is associated with sporadic cases and minor localized outbreaks. As influenza C poses much less of a disease burden than influenza A and B, only the latter two are included in seasonal influenza vaccines.[3]

Influenza A and B viruses are important human respiratory pathogens which are transmitted mainly by droplets and aerosols originating from the respiratory secretions of infected people, but occasionally also through contact with virus contaminated fomites.[7] In temperate climates, disease tends to occur seasonally in the winter months, spreading from person-to-person through sneezing, coughing, or touching contaminated surfaces.[3]

Influenza occurs globally with an annual attack rate estimated at 5%–10% in adults and 20%–30% in children. In temperate climates, seasonal epidemics are experienced mainly during the winter while in tropical regions, influenza may occur throughout the year, causing outbreaks more irregularly. The morbidity and mortality from influenza are likely to be underestimated in the tropics and subtropics. A systematic review covering 30 years of seasonal influenza epidemiology in sub-Saharan Africa

[5]Pretty Multiharina et al, Laboratory Network in ILI and SARI Surveillance System for Influenza Virus Evolution Monitoring, IIF Symposium 2013.

[6]Pink Book, Chapter: Influenza, Center for Disease Control.www.cdc.gov/vaccines/pubs/pinkbook/index.html,downloadedJuly 2016.

[7]WHO, Weekly epidemiological record, Vaccines against influenza WHO position paper-November 2012, Geneva 2012, 47(87); 461-476

showed that on average, influenza accounted for about 10% (range 1%–25%) of all outpatient visits and for about 6.5% (range 0.6%–15.6%) of hospital admissions for acute respiratory infections in children.[7]

Continual antigenic drift of the influenza virus means that a new vaccine, updated yearly with the most current circulating strains, is needed to protect against new infections. Each 0.15 µg of hemagglutinin of each antigen. The vaccine will be available as a split/sub unit virus preparation. Both humoral and cell mediated responses are thought to play a role in immunity to influenza. Immunity declines over the year following vaccination.[8]

Influenza is an under appreciated contributor to global mortality and morbidity and has significant economic consequences. Current estimates indicate that each year, seasonal influenza affects 5-10% of the world's population resulting in between 250.000 and 500.000 deaths.[9] In South East Asia, there was a decreasing trend in influenza detection in recent weeks, although some countries in the region reported ongoing activity of co-circulating seasonal influenza A and B viruses.[10] In Indonesia, the Influenza cases were range between 15-20% for Influenza A and 5-10% for Influenza B and it happened all of the year.[5]

Children aged < 5 years, and particularly those < 2 years of age, have a high burden of influenza. A systematic review of the global disease burden of influenza in children, representing studies on a total od around 8 million children < 5 years of age, estimated that in 2008, there were 90 million new cases of seasonal influenza, 20 million cases of influenza-associated acute lower respiratory infections (ALRI), and 1-2 million cases of influenza asociated severe ALRI including 28.000-111.500 deaths.[7] The great majority of death from influenza occurred in developing countries.[11]

8 Fukuda K, Levandowski RA, Bridges CB et al. Inactivated influenza vaccines. In Plotkin SA, Orenstein WA, with assistance of Offit PA. vaccines, Saunders: Philadelphia, 2004: 339-700.

[9] World Health Organization. A Manual for Estimating Disease Burden Associated with Seasonal Influenza. Geneva: World Health Organization; 2015.

[10] World Health Organization. Influenza Update – 273. Geneva: World Health Organization; 2016 [cited 2016 October 12]. Available from: http://www.who.int/influenza/surveillance_monitoring/updates/latest_update_GIP_surveillance/en/

[11] Nair H, et al. Global burden of respiratory infections due to seasonal influenza in young children; a systemic review and meta analysis. The Lancet 2011, 378:1917-30.

1.3. Prevention and Control of Infection among Humans

Vaccination is the most effective way to prevent infection and severe outcomes caused by influenza viruses. Development and production of influenza vaccines, planning for their supplies and use as well as provision of other respective health care resources are essential components of a comprehensive seasonal and pandemic influenza response.[12] Internationally available vaccines for the control of seasonal influenza are safe and efficacious and have the potential to prevent significant annual morbidity and mortality. Most of the current seasonal influenza vaccines include 2 influenza A strains and 1 influenza B strain.[7]

Quadrivalent influenza vaccines that could potentially provide wider protection against influenza B viruses are becoming available and recommendations should not be limited to trivalent vaccine formulations.[7]

The quadrivalent flu vaccine is designed to protect against four different flu viruses; two influenza A viruses and two influenza B viruses. For years, flu vaccines were designed to protect against three different flu viruses (trivalent). This included an influenza A H1N1 virus, an influenza A H3N2 virus and one B virus. Experts had to choose one B virus, even though there are two very different lineages of B viruses that both circulate during most seasons. This meant the vaccine did not protect against the group of B viruses not included in the vaccine. Adding another B virus to the vaccine aims to give broader protection against circulating flu viruses.[13]

1.4. Rationale

Although influenza vaccination aims primarily at protecting vulnerable high risk groups against severe influenza-associated disease and death, influenza causes considerable morbidity worldwide even beyond these groups and therefore represents a public health problem with significant socioeconomic implications.[7]For countries considering the initiation or expansion of programmes for seasonal influenza

[12] World Health Organization. Vaccines. Geneva: World Health Organization [cited 2016 October 12]. Available from: http://www.who.int/influenza/vaccines/en/

[13] Center for Disease Control and Prevention. Quadrivalent Influenza Vaccine. Atlanta: Center for Disease Control and Prevention; 2016 [cited 2016 October 12]. Available from: http://www.cdc.gov/flu/protect/vaccine/quadrivalent.htm

vaccination, WHO recommends that pregnant women should have the highest priority. Additional risk groups to be considered for vaccination, in no particular order of priority, are children aged 6–59 months, the elderly, individuals with specific chronic medical conditions, and health-care workers. Countries with existing influenza vaccination programmes targeting any of these additional groups should continue to do so and should incorporate immunization of pregnant women into such programmes.[7]
One dose of Inactivated Influenza Vaccine (IIV) may be administered annually for persons 9 years of age or older. Children 6 months through 8 years of age receiving influenza vaccine for the first time should receive two doses administered at least 28 days apart.[6]
At first, Bio Farma formulated trivalent inactivated influenza vaccine but in 2016 Bio Farma starts to formulate the quadrivalent inactivated influenza vaccine because it will give a broader protection against circulating flu viruses.

1.5 Previous Study

Bio Farma conducted several study for seasonal trivalent influenza vaccine but no for quadrivalent influenza vaccine.
From August to November 2008, a total of 405 adolescents and adults were enrolled for this bridging study of seasonal influenza HA vaccine (formulated in Bio Farma). The vaccine induced high antibody titers against influenza antigens in adolescent and adults, the percentage of subjects with anti-influenza titer ≥ 1:40 HI units to A/Hiroshima, A/Solomon Island and B/Malaysia strain were not different (97.8%; 98.2%, and 95.5%, respectively; p=0.025). The Geometric Mean Titer (GMT) after immunization were increased (A/Hiroshima, GMT: 66.16 to 323.37; A/Solomon Islands, GMT: 41.89 to 554.26; and B/Malaysia, GMT: 24.02 to 231.83). Percentage of subjects with increasing antibody titer > 4 times of anti A/Hiroshima, A/Solomon, and B/Malaysia were: 61.2%; 85.5%; and 81.5%, respectively. The percentage of subjects with transition of seronegative to seropositive of anti A/Hiroshima, A/Solomon, and B/Malaysia were 93.7%, 95.8%, and 93.9%, respectively. There were 81 (20.0%) who reported with local reaction and 16.3% who reported with

systemic reaction. All vaccines were well-tolerated and no serious adverse events during the study.[14, 15]

A yearly licensing study was conducted in 2010. From January to February 2010, a total of 60 adolescents and adults were enrolled for yearly licensing study (Phase IV). The percentage of subjects with anti-influenza titer ≥ 1:40 HI units to A/Brisbane/59/2007 (H1N1), A/Uruguay/716/2007 (H3N2), and B/Brisbane/60/2008 strain were high (100.0%; 100.0%, and 100.0%, respectively; p=1.000). The GMT of anti A/Brisbane/59/2007 (H1N1), A/Uruguay/716/2007 (H3N2), and B/Brisbane/ 60/2008 titers 28 days after immunization was 738.5 HI units; 420.0 HI units; and 264.3 HI units, respectively. Percentage of subjects with increasing antibody titer > 4 times, 28 days after immunization of anti to A/Brisbane/59/2007 (H1N1), A/Uruguay/716/2007 (H3N2), and B/Brisbane/60/2008 were: 75.8%; 84.5%; and 77.6%, respectively. The percentage of subjects with transition of seronegative to seropositive of anti to A/Brisbane/59/2007 (H1N1), A/Uruguay/716/2007 (H3N2), and B/Brisbane/60/2008 were 100.0%; 100.0%, and 100.0%, respectively. All vaccines were well-tolerated. There were no serious adverse events reported during the study.[16]

A similar study was conducted in 2011. Healthy adolescents and adults (12–61) years were assigned to receive one dose (0.5 ml) intramuscular injection of trivalent recombinant hemagglutinin containing H1, H3 and B antigens (Ag) derived from 2009 to 2010 influenza virus strains A/California/7/2009 (H1N1), A/Victoria/210/2009 (H3N2), B/Brisbane/60/2008. From January to February 2011, a total of 61 adolescents and adults were enrolled. The vaccine induced high antibody titers against influenza antigens in adolescents and adults, the percentage of subjects with anti-influenza titer ≥ 1:40 HI units to A/California/7/2009 (H1N1), A/Victoria/210/2009 (H3N2), B/Brisbane/60/2008 strain were not different (100.0%; 100.0%, and 100.0%, respectively; p=1.00). The Geometric Mean Titer (GMT) after

[14]Dhamayanti M, Rusmil K, Idjradinata P. Respon Imun terhadap Vaksin Influenza pada Remaja. Jurnal Kedokteran Brawijaya 2012, 27(2):102-6.

[15]Rusmil K, Fadlyana E, Dhamayanti M, Gunadi R, Bachtiar NS. Protectivity and Safety of Influenza HA Vaccine (PT Bio Farma) in Adolescents and Adults**.** Poster Presentation Indonesian Influenza Foudation Symposium 2013.

[16]Fadlyana E, Rusmil K, Bachtiar NS, Gunadi R, Sukandar H. Immunogenicity and safety of a trivalent inactivated influenza vaccine. Paediatrica Indonesiana 2011;51(1):22-8.

immunization were increased (A/California/7/2009 (H1N1), GMT: 23.24 to 394.00; A/Victoria/210/2009 (H3N2), GMT: 44.90 to 394.37; and B/Brisbane/60/2008, GMT: 34.03 to 269.09). Percentage of subjects with increasing antibody titer > 4 times, 28 days after immunization of anti A/California/7/2009 (H1N1), A/Victoria/210/2009 (H3N2), B/Brisbane/60/2008 were: 93.3%; 80.0%; and 78.3%, respectively. The percentage of subjects with transition of seronegative to seropositive of anti A/California/7/2009 (H1N1), A/Victoria/210/2009 (H3N2), B/Brisbane/60/2008 were 100.0%, 100.0%, and 100.0%, respectively. All vaccines were well-tolerated.[17]

A study using influenza vaccine produced by Biken institute was conducted in 2005 to children < 4 years of age. 259 children were involved received 2 doses of vaccines with the interval 4 weeks. Seroprotection were achieved by 23-42% in 0 year old, 49- 58% in 1 year old, 67-89% in 2 years old and 71-85% in 3 years old children. Immune response against influenza vaccine was highly influenced by prevaccination antibody levels and age.[18]

A study using influenza vaccine Flubio® was conducted in 2014 to children 6 months – 11 years old in Jakarta. Four houndred and four children were involved receiving 1 or 2 doses of vaccines. The seroprotection rate of Influenza HA vaccine (anti Influenza titer > 1:40 HI) 28 days after immunization in infants and children in all age groups reached 100% for all strains. However, percentage of subjects with increasing antibody titer ≥ 4 times in each group 28 days after immunization was not sufficiently high. Group A (6 – 35 months) percentage of subjects with increasing antibody titer > 4 times only increased 63.4% for A/California, 67.25% for A/Texas, and 67,2% for B/Massachusets. Percentage subjects in group C (9-11 years old), for strain A/Texas only increased 36.6%. This result needs more study to find out how long the antibody persistence against influenza in group A.[19]

---

[17]Fadlyana E, Rusmil K, Bachtiar NS, Gunadi R, Sukandar H. Immunogenicity and safety of a trivalent inactivated influenza vaccine. Poster Presentation Indonesian Influenza Foudation Symposium 2013.

[18]Irie S, Fujieda M, Ito K, Ishibashi M, Takamizawa T, Ishikawa T,et al. Immune response against inactivated influenza vaccine in children under four years old. The Journal of Infectious Disease, 2007:81;284-90.

[19]Medise BE, Gunardi H, Sekartini R, Soedjatmiko, Satari HI. Immunogenicity and Safety of Flubio (Influenza HA) Vaccine in Infants and Children (Bridging Study). Department of Child Health, School of Medicine, University of Indonesia. 2015 (not published)

A study using influenza HA vaccine (BIKEN HA, FLUBIK HA, FLUBIK HA Syringe) was conducted in Japan to evaluate the safety and immunogenicity when two doses of BK-FLU (Influenza HA vaccine (quadrivalent)) were subcutaneously administered into Japanese adults. A total of 55 subjects involved in this study. When two doses of BK-FLU were subcutaneously administered, side reactions were reported in 47 cases out of 55 cases (85.5%). Local reactions at the injection site and systemic side reactions were reported in 47 cases (85.5%) and 8 cases (14.5%) respectively. Nevertheless, no serious adverse events were reported. When the positive conversion ratio of HI antibody titer and neutralization antibody titer, the GMP change rate, and the antibody prevalence rate were calculated, the production of antibody to all the four strains was observed, including subtype A (H1N1) strain, subtype A (H3N2) strain, subtype B (Yamagata lineage) strain, and subtype B (Victoria lineage). It is considered that BK-FLU is safe when 0.5 mL of this investigation product is subcutaneously administered into Japanese healthy adults at the age of not less than 20 years and less than 65 years old twice at an interval of 1 to 4 weeks. Besides, it is confirmed that BK-FLU produces the antibody to all the four strains was observed, including subtype A (H1N1) strain, subtype A (H3N2) strain, subtype B (Yamagata lineage) strain, and subtype B (Victoria lineage).[20] Based on this study, quadrivalent influenza HA vaccine which produced by BIKEN has been registered in Japan.

[20] The Research Foundation for Microbial Diseases of Osaka University. Clinical Trial for BK-FLU on Healthy Adults. (not published)

2. Objectives

2.1. Primary objective

To assess the protectivity rate of Quadrivalent Influenza HA vaccine 28 days after immunization in Indonesian population.

2.2. Secondary Objectives

- To describe immunogenicity of quadrivalent Influenza HA vaccine in all subjects
- To assess the safety of quadrivalent Influenza HA vaccine in all subjects
- To evaluate immunogenicity and safety one dose of quadrivalent influenza HA vaccine compare to trivalent Influenza HA vaccine in group 9-40 years of age.
- To evaluate immunogenicity and safety in three consecutive batches of quadrivalent Influenza HA vaccine in group 9-40 years of age.

3.1. Trial Design

This Quadrivalent Influenza HA vaccine (Bio Farma) was formulated in Bio Farma. The bulk was imported from Japan. The clinical data in Japan were sufficient to support the study in Indonesia. The aim of this study is to assess the antibody response influenza hemagglutination which formulated in Bio Farma in Indonesian population.

There are two sub-studies in this protocol

- **Sub Study A (Subjects 9 - 40 years of age) :**

To evaluate immunogenicity and safety of quadrivalent compare to trivalent Influenza HA vaccine and to evaluate lot to lot consistency in three consecutive batches of quadrivalent Influenza HA vaccine. This is an experimental, randomized, double blind bridging study.

Five hundred forty (540) subjects will be involved in this study. Each subject will receive one dose of quadrivalent influenza vaccine with different batch number (batch 1/batch 2/batch 3) or trivalent HA vaccine according to the randomization list. Each group consist of 180 subjects, @ 45 subjects will receive QIV batch 1/2/ 3 or TIV.

Group	Age	Vaccine				Vaccine dose
		**QIV1**	**QIV2**	**QIV3**	**TIV**	
**I**	**9 – 12 years**	**45**	**45**	**45**	**45**	**0.5 ml**
**II**	**13– 17 years**	**45**	**45**	**45**	**45**	**0.5 ml**
**III**	**18 – 40 years**	**45**	**45**	**45**	**45**	**0.5 ml**
**Total Subjects**		**135**	**135**	**135**	**135**	**-**

The subject will be grouped into three different batch of Quadrivalent Influenza vaccine to evaluate lot to lot consistency batches.

- **Sub Study B (Subjects 6 months - 8 years of age)**

This is an experimental, open labelled bridging study.

270 infants and children will be involved in this study. Each subject will receive two doses of influenza HA vaccine from one batch number.

Group	Vaccine	Age	Number of subjects
IV	3070217	6-35 Months	135
V	3070217	3-8 Years	135

3.2. Treatment Allocation Procedures

Sub-study A

After being informed about the study, collecting a signed informed consent from the subjects or signed assent and informed consent (for subjects 9-17 years old), the investigator will check inclusion and exclusion criteria. For each subject recruited, inclusion number will be allocated in the chronological order of the subject which include in the trial from I001 to I180 (for group 9-12 years), II001-II180 (for group 13-17 years), III001-III180 (for group 18-40 years)).

The subject will be randomized per treatment group.

The doctor will strictly follow the list of randomization provided by Bio Farma. Treatment will be allocated in accordance with a randomization list, so that to each randomization number, corresponds only one strictly randomly assigned treatment group (A/B/C/D).

Sub-Study B

After being informed about the study, collecting a signed informed consent from the parents, the investigator will check inclusion and exclusion criteria. For each subject recruited, inclusion number will be allocated in the chronological order of the subject which include in the trial from IV001 to IV135 (for group 6-35 months) and V001-V135 (for group 3-8 years).

3.3. Trial Population

3.3.1. Inclusion Criteria

Subjects 9 - 40 years of age

1. Healthy
2. Properly informed about the study and having signed the informed consent form
3. Subject/Parent will commit themselves to comply with the instructions of the investigator and the schedule of the trial.

Subjects 6 months - 8 years of age:

1. Healthy
2. Parents have been informed properly regarding the study and signed the informed consent form
3. Parents will commit themselves to comply with the instructions of the investigator and the schedule of the trial

3.3.2. Exclusion Criteria

1. Subject concomitantly enrolled or scheduled to be enrolled in another trial
2. Evolving mild, moderate or severe illness, especially infectious diseases or fever (axillary temperature ≥ 37.5°C)
3. Known history of allergy to egg and or chicken protein or any component of the vaccines
4. History of uncontrolled coagulopathy or blood disorders contraindicating intramuscular injection
5. Subject who has received in the previous 4 weeks a treatment likely to alter the immune response (intravenous immunoglobulins, blood-derived products or long term corticotherapy (> 2 weeks).
6. Pregnancy & Lactation (Adult)
7. Any abnormality or chronic disease which according to the investigator might interfere with the assessment of the trial objectives
8. Subject already immunized with influenza vaccine within 1 year.
9. Subject receives any vaccination within 1 month before and after immunization of Quadrivalent Influenza Vaccine.

3.3.3. Prior and Concomitant Therapy

Treatments that are forbidden during the trial: Any treatment such as, but not limited to, intravenous immunoglobulins, systemic corticosteroid or blood products, susceptible to alter the immune response. In this case, 4 weeks are required between previous treatment stop date and administration of investigational vaccine(s).

Forbidden concomitant vaccinations: other Influenza vaccines.

For all treatments mentioned above and other therapies taken during the trial, the following items should be reported on the CRF:

- Trade name
- Total daily dose
- Route of administration
- Start and stop dates of administration
- Indication. The investigator should use acceptable medical terminology to detail the reason for prescription.

3.4. Trial Plan

3.4.1. Conditions for conducting the trial

3.4.1.1. Ethical Considerations/Protocol Review

Protocol review

Before the inclusion of the first subject in the center, the protocol should be signed by the investigators and sponsor's representatives and approved by the Quality Assurance Division of Bio Farma, by the Institutional Ethics Committee and Indonesian Regulatory Authorities.

Ethical Considerations

This trial will be conducted in accordance with the latest Edinburg, Scotland revision of the Declaration of Helsinki, ICH Good Clinical Practice guidelines and local regulatory requirements.

The investigator shall be responsible for obtaining approval of the protocol from the Institutional Ethics Committee before start of the trial, as well as approval of all amendments in compliance with local law. Copies of these approvals must be forwarded by the investigator to Bio Farma with the composition (names and qualification of the members) of the Institutional Ethics Committee.

3.4.1.1.1. Informed Consent/Assent

The investigator or delegate must obtain the written informed consent/and assent from the subject or parent/guardian(s) before any study-related procedures are performed and after the subjects have been informed of the nature of the trials, the potential risks and his/her obligations.

Written informed consent should given by the subject 18-40 years of age, or by the parents/guardian (s) for children (6 month-8 years of age). While the subject 9-17 years of age, should give his/her written assent before their parents or guardian(s) give written informed consent.

3.4.1.1.2. Subject Benefits/Potential Risks

The subjects enrolled in this trial would potentially gain benefit from vaccination with an Influenza HA vaccine. As to the risks, refer to the package insert of Influenza HA vaccine.

3.4.1.2. Modification of the protocol

No amendments to this protocol will be made without consultation with and agreement of the sponsor. Any amendment to the trial that seems indicated as the trial progresses must be discussed by the investigator and sponsor concurrently. If agreement is reached concerning the need for an amendment, such amendment will be produced in writing by the sponsor and/or the investigator and be made a formal part of the protocol.

An amendment requires Institutional Ethics Committee approval. It should also be transmitted to Indonesian Regulatory Authorities, if applicable.

An administrative change to the protocol is one that modifies administrative and logistic aspect of a protocol and that does not affect the subject safety, the objectives of the trial and its progress. An administrative change only requires Institutional Ethics Committee notification.

The investigator is responsible for insuring that changes in approved trial, during the period for which Institutional Ethics Committee approval has already been given, may not be initiated without Institutional Ethics Committee review and approval except where necessary to eliminate apparent immediate hazards to the human subjects.

3.4.1.3. Interruption of the Trial

The trial may be discontinued for administrative reasons, if new data about the investigational product(s) resulting from this trial become available, and/or on advice of the sponsor, the investigators and/or the Institutional Ethics Committee.

If a trial is prematurely terminated or suspended, the sponsor shall promptly inform the investigators, the Indonesian Regulatory Authorities and the Institutional Ethics Committee of the reason for termination or suspension.

3.4.2. Trial Calendar/Timelines

Recruitment	: October - December 2017
End of Follow-up	: March 2018
Serology testing	: April - June 2018
Data Management	: October 2017 – March 2018
Statistical analysis	: June - July 2018
Preliminary Report	: August - September 2018
Clinical Report	: October 2018

3.4.3. Trial Center

This trial will be implemented in one center, Department Of Child Health Hasan Sadikin General Hospital/Medicine Faculty Padjadjaran University Bandung, West Java.

The subjects will be recruited in:

- Hasan Sadikin General Hospital, Bandung, West Java, Indonesia
- Puskesmas Ibrahim Adjie (Primary Health Center)
- Puskesmas Puter (Primary Health Center)
- Puskesmas Garuda (Primary Health Center)

3.4.4. Vaccination and Serology Schedule

Vaccination Schedule	Serology Schedule
**Subjects 9- 40 years of age**<br>V1 (Day 0) : 1st dose of Influenza HA Vaccine	**Subjects 9 - 40 years of age**<br>V1 (Day 0) : before immunization<br>V2 (Day 0 + 28 (-4/+7)) days
**Subjects 6 – 35 months of age and 3-8 years of age:**<br>- V1 (Day 0) : 1st dose of Influenza HA vaccine<br><br>- V2 (Day 0 + 28 (-4/+7)days): 2nd dose of Influenza HA vaccine	**Subjects 6 months - 8 years of age**<br><br>- V1 (Day 0) : before immunization<br>- V3 (Day 0 + 56 (-4/+7)) days

3.4.5. Procedure for Obtaining, Handling and Shipment of Serum Samples

3.4.5.1. Obtaining Serum Samples

Four ml of blood will be collected in vacutainer tubes at visit V1, V2 (group I-III) and V3 (group IV & V). The person in charge of blood drawing should verify the subject's identity and should check that the initials on the laboratory request are those of the subject just before taking the blood sample.

Then, he/she should write the subject's initials on all labels of the corresponding band. He/she should affix one label onto the Vacutainer tube immediately prior to blood sample drawing. It is absolutely necessary to obtain a sterile blood sample.

After clotting at room temperature from 30 minutes to 2 hours, blood samples will be centrifuged at 3000 rpm for 15 minutes and the sera will be separated aseptically into 2 aliquots (1.5 ml each) within 24 hours after the sampling. First aliquot will be tested for the serology screening before recruitment. Second aliquot will be stored as the pre immunization samples to be tested for antibody titer after the blinding procedure. Sera will be rapidly stored in a freezer at –20°C/–80°C pending collection.

Each blood sample will be labeled with sticker, which indicates the blood sampling stage (V1, V2 and V3), the trial code, the inclusion number and the subject's initials. The labels will mention neither the group nor the vaccine injected to allow the blinding of serologies.

3.4.5.2. Handling Serum Samples

3.4.5.2.1. Storage Conditions

Each sample properly labeled should be frozen at –20°C to –80°C. Temperature should be monitored and documented on the appropriate form during the entire trial.

3.4.5.2.2. Blinding Procedure

The serology testing will be started only after the samples had been blinded. The blinding code and list will be prepared by the statistician. The blinding procedure will be witnessed by the investigator and team. The samples were stored in the cryo box according to the new code. The key of the blinding code will be kept by the statistician and the investigator. The blinding code will be opened after the result of serology testing had been received by the investigator.

3.4.5.2.3. Method and Timing of Measurement

Antibody titers are measured at visit V1 (Day 0) and at visit V2 or V3. These titers will be evaluated by the following assay technique:

Antigen	Methods	Units
Influenza Hemagglutination	Hemagglutination Inhibition test	HI unit

Any additional serological analysis on antigen(s) included in the vaccine tested in this trial may be performed if Bio Farma considers it necessary to further document the immunogenicity results of this trial or other trials.

HI assays will be performed in Immunology Laboratory of Clinical Trial Department of Bio Farma accordance with standard methods from Biken Vaccine Institute. This HI method had been validated and approved by Quality Asurance Division.[13]

In U bottomed 96 well plates, 25µl antigens from same strains of the vaccine with HA titer of 8 HA units is mixed with 25µl of two fold dilution RDE-treated serum in PBS. After 60 minutes of incubation in room temperature, 50µl of O human red blood cells in added to the mixtures. The titer is defined as the highest dilution of the serum which is able to inhibit hemagglutination.[14]

3.5. Case Report Form and Data Collection

All information will be recorded by the investigator or a designated person in the Case Report Forms provided by Bio Farma. They will be filled out with a black ball- point pen, in capital letters and signed by the investigator.

Explanations must be given for all missing information. All incorrect data must be crossed out with a single line, then signed and dated by the investigator (except date corrections). “White-out” correction fluid should not be used.

3.6. Subject Diaries and Interim Histories

Subjects will keep an observation card (diary) to assess and record information for local/systemic reactions for 28 days following immunization with special attention within the first three days. Safety data within 3 days after immunization (V1+3 days) will be followed up by phone.

The local reaction assessment will involve assessment of the vaccination site. The systemic event assessment will involve daily axillary temperature readings (subject/parent will be supplied with a thermometer and instructed how to use it) and recording any systemic complaints such as weakness on a diary. Any medical office

visit, emergency room visit or hospitalization for any reason will be recorded throughout the trial period. Moreover, any serious adverse event occurring throughout the trial period should be reported immediately and will also be recorded in the CRF.

3.7. Clinical Supplies

Protocols, CRFs, and diaries will be provided by Bio Farma.

4. Products

4.1. Investigational Product Characteristics: Quadrivalent Influenza HA vaccine

The current inactivated quadrivalent influenza vaccine contains 4 hemagglutination of four strains of influenza vaccine.Type A/H1N1, A/H3N2, and Type B. In this trial, Influenza HA vaccine formulated from 4 bulk of monovalent influenza vaccine; A/California/7/2009 (X-179A)(H1N1)pdm09, A/Hong Kong/4801/2014(X-263) (H3N2) , B/Texas/2/2013 and B/Phuket/3073/2013.
Because of the frequent emergence of new influenza variant strains, the antigenic composition of influenza vaccines needs to be evaluated yearly, and the inactivated influenza vaccines are reformulated almost every year.

4.1.1. Product Description

Quadrivalent Influenza HA vaccine contains 4 strains of influenza antigens..

4.1.2. Composition

Each ml is composed of (one dose corresponds to 0.5 ml):

**Form**	Liquid in vial of single dose
**Dose**	0.5 ml (15 µg HA for each strain*, thimerosal 4µg)
**Route**	Intramuscular injection
**Batch Number**	3070117, 3070217, 3070317
**Expired Date**	February 2018

*List of Strains:
A/California/7/2009 (X-179A)(H1N1)pdm09
A/Hong Kong/4801/2014(X-263) (H3N2)
B/Texas/2/2013
B/Phuket/3073/2013

4.2 Control Product Characteristics: Trivalent Influenza HA vaccine

The inactivated trivalent influenza vaccine contains 3 hemagglutination of three strains of influenza vaccine.Type A/H1N1, A/H3N2, and Type B. In this trial, Influenza HA vaccine formulated from 3 bulk of monovalent influenza vaccine; A/California/7/2009 (X-179A)(H1N1)pdm09, A/Hong Kong/4801/2014(X-263) (H3N2), B/Texas/2/2013. Because of the frequent emergence of new influenza variant strains, the antigenic composition of influenza vaccines needs to be evaluated yearly, and the trivalent inactivated influenza vaccines are reformulated almost every year.

4.2.1. Product Description

Influenza HA vaccine contains 3 strains of influenza antigens.

4.2.2. Composition

Each ml is composed of (one dose corresponds to 0.5 ml):

**Form**	Liquid in vial of single dose
**Dose**	0.5 ml (15 µg HA for each strain*, thimerosal 4µg)
**Route**	Intramuscular injection
**Batch Number**	3020117
**Expired Date**	February 2018

* List of strains:

– A/California/7/2009 (X-179A)(H1N1)pdm09
– A/Hong Kong/4801/2014(X-263) (H3N2)
– B/Texas/2/2013

4.3. Preparation

Shake well the product before injection.

4.3.1. Precautions for use

The vaccine must be stored at +2°C to +8°C and should not be frozen.The skin at the sites of injection will be cleaned and disinfected prior to injection. The vaccinator will use a separate sterile syringe for each individual trial part. The vaccines will be injected intramuscularly into the **left antero lateral thigh region for subjects ≤ 2 years or left deltoid region for subjects > 2 years**, by inserting the needle with a brisk dart-like action at a right angle to the skin surface. The needle should be inserted to its full length, and the vaccinator should take care not to inject the vaccine into a blood vessel. In case this occurs, injection should be repeated using the same procedure at another site.

4.3. Administration

The vaccines will be injected intramuscularly into the left antero lateral thigh region for subjects ≤ 2 years or left deltoid region for subjects > 2 years.

Schedule:
One dose of Quadrivalent or Trivalent Influenza Vaccine at visit 1 (Day 0) for group I, II, III
One dose of Quadrivalent Influenza Vaccine vaccine at at visit 1 (Day 0) and visit 2 (Day 0+28 days-4/+7 days) for group IV & V.

4.4. Labeling and Packaging

Sub-study A

Vaccines will be supplied in single dose vial. Special blinding label will be used for each investigational product and control. The information on the label will be coded, except expired date, instruction of storage and route of administration. Additional label "**hanya untuk uji klinis**" (for clinical study only) will be added to each label. The vaccine code will be determined by the Pharmaceutical Production Division of Bio Farma and is confidential.

Sub-study B

Vaccines will be supplied in single dose vial. The quadrivalent influenza vaccine which has not been registered yet, generic label will be affixed to the vial and mentions "**hanya untuk uji klinis**" (for clinical trial only).

4.5. Storage and Shipment Conditions

4.5.1. Shipment Conditions

Influenza HA vaccines will be provided by Bio Farma. Products will be sent to the center according to this pre-determined schedule. For each vaccine transfer, the person in charge of product receipt will check that the cold chain was maintained during the transfer. In case of problem, he/she should alert the Monitor immediately.

4.5.2. Storage Conditions

Vaccines shall be stored at a temperature ranging from +2°C to +8°C (in a refrigerator). Temperature should be monitored and documented on the appropriate form (see operating guidelines) during the entire trial.

In case of deep freezing or accidental disruption of the cold chain, vaccines should never be administered and the investigator or the responsible person should contact the Monitor to receive further instructions.

4.6. Accountability

Products should be kept in a secure place.The investigator or the person in charge of product management should maintain records of the product's delivery to the trial site, the inventory at the site, the dose(s) given to **each subject (1 vial for each subject)** and the return of unused doses to the sponsor.

4.7. Return of Unused Products

Unused and/or open products will be returned to Bio Farma at the end of the vaccination period together with the form “Return of unused and/or open products” in accordance with the Monitor’s instructions.

5. Trial Administration

5.1. Personnel involved in the trial

**Principal Investigator**	Dr. Meita Dhamayanti, dr.,Sp.A(K).,MKes.
**Medical Advisor**	Prof. Cissy B. Kartasasmita, dr.,Sp.A(K).,MSc.,PhD.
**Subinvestigators**	1. Prof. Dr. Kusnandi Rusmil, dr.,Sp.A(K).,MM.<br>2. Dr. Eddy Fadlyana, dr.,Sp.A(K).,MKes.<br>3. Rodman Tarigan, dr.,Sp.A(K).,Mkes.<br>4. Susantina Prodjosoewojo, dr.,Sp.PD<br>5. Andri Reza Rahmadi, dr.,Sp.PD.,MKes.
**Biometry**	Dr. Drs. Hadyana Sukandar, MSc.
**Biological Laboratory**	Rini Mulia Sari, dr.<br>Yani Sukriyani<br>Restika Andiarini
**Monitor**	Dr.Novilia Sjafri Bachtiar, dr., MKes.<br>Rini Mulia Sari, dr.<br>Julianita Fahmi, dr.<br>Asep Irham, F. Q., dr

5.2. Visit Procedures

<u>Sub Study A (Group I-III)</u>

<u>Visit 1.</u> (D0) 1st blood sample & 1st vaccination

a. Provide subject or parent/guardian(s) with the relevant information concerning the trial.
b. Obtain informed consent/and assent, dated and signed by the subject/parents.
c. Check eligibility criteria.
d. Perform a physical examination.
e. Allocate an inclusion number and randomization code, chronologically to the enrolment of the subject.
f. Take a pre-vaccination blood sample (V1, 4 ml).
g. Inject the 1st dose of the quadrivalent or trivalent influenza vaccine
h. Put the initial name and the inclusion number of the subject on the used vial.

i. Record the code of vaccine in the CRF and the list of participant.
j. Keep the subject under observation for 30 minutes and evaluate the immediate local reactions and systemic events.
k. Complete CRF.
l. Provide a diary card (DC1) to the subject or parent/guardian (s) and inform them how to use and fill the diary card.
m. Inform the subject/ parent that they should contact the investigator or the study nurse in case of any Serious Adverse Event.
n. Instruct the subject/ parent to return for visit 2.

⇒ **<u>Visit 2 (V1 + 28 (-4/+7) days)</u> 2nd blood**

a. Perform a physical examination
b. Check the possible Serious Adverse Events, which occurred since the last visit.
c. Record the safety data from DC1 to CRF.
d. Check the concomitant therapies and record in the CRF.
e. Take a blood sample (V2, 4 ml)
f. Completion of the study.

<u>Sub Study B (Group IV-V)</u>

<u>Visit 1.</u> (D0) 1st blood sample & 1st vaccination

a. Provide parent/guardian(s) with the relevant information concerning the trial.
b. Obtain informed consent, dated and signed by the parent/guardian(s).
c. Check eligibility criteria.
d. Perform a physical examination.
e. Allocate an inclusion number chronologically to the enrolment of the subject.
f. Take a pre-vaccination blood sample (V1, 4 ml).
g. Inject the 1st dose of the quadrivalent influenza vaccine
h. Put the initial name and the inclusion number of the subject on the used vial.
i. Record the batch of vaccine in the CRF and the list of participant.
j. Keep the subject under observation for 30 minutes and evaluate the immediate local reactions and systemic events.
k. Complete CRF.
l. Provide a diary card (DC1) to the subject or parent/guardian (s) and inform them how to use and fill the diary card.
m. Inform the parent/guardian(s) that they should contact the investigator or the study nurse in case of any Serious Adverse Event.
n. Instruct the parent/subjec (s) to return for visit 2.

⇒ **<u>Visit 2 (V1 + 28 (-4/+7) days)</u> 2nd injection**

a. Perform a physical examination

b. Check the possible Serious Adverse Events, which occurred since the last visit.
c. Record the safety data from DC1 to CRF.
d. Check the concomitant therapies and record in the CRF.
e. Inject the 2nd dose of the quadrivalent influenza vaccine.
f. Put the initial name and the inclusion number of the subject on the used vial.
g. Record the batch of vaccine in the CRF and the list of participant.
h. Keep the subject under observation for 30 minutes and evaluate the immediate local reactions and systemic events.
i. Complete CRF.
j. Provide the diary card (DC2) to the parents, and inform them how to use and fill the diary card.
k. Inform the subject's parents that they should contact the investigator or the study nurse in case of any Serious Adverse Event.
l. Instruct the parents to return for visit 3.

⇒ <u>Visit 3.</u> (V2+28 days (-4/+7) days) 2nd blood sample

a. Perform a physical examination.
b. Check the possible Serious Adverse Events, which occurred since the last visit.
c. Record the safety data from DC2 to CRF.
d. Check the concomitant therapies and record in the CRF.
e. Take a post-vaccination blood sample (V3, 4 ml).
f. Complete CRF.
g. Completion the study.

5.3. Conditions for Withdrawal from the Trial

The investigator may decide to discontinue the treatment (i.e. vaccination), should an event which is considered as a definite contra-indication, is occured during the trial.

5.4. Lost to Follow-up Procedures

In case of subjects failed to visit for a follow-up examination, extensive effort should be taken to locate or recall them or at least to determine their health status. These efforts should be documented in the subject's CRF and source documents.

5.5. Termination Classification

Definitions:

- <u>Discontinuation by the investigator:</u> an observation is considered a discontinuation by the investigator when the latter decides to terminate the subject's participation for medical reasons (for subject's safety), for personal reason (it is his opinion that the subject can not continue to participate), etc.

- Drop-out: a subject included in the trial is said to have dropped out after deciding, on his own volition, to terminate his participation in the trial. Subjects may decide to withdraw their subject from the trial at any time. The investigator should makesure, however, that withdrawal was not due to an adverse event. The reason for withdrawal should be noted in the space provided for this purpose in the CRF.
- Lost to follow-up: the subject could not be found in spite of the investigator's researches.
- Death.

5.6. Monitoring, Auditing and Archiving

5.6.1. Routine Monitoring

5.6.1.1. Set-up Visit

A set-up visit will be performed before the inclusion of the first subject in the center. The monitor and/or the Medical Responsible will verify and document that the material to be used during the trial has been received and that the investigational team has been properly informed about the trial, regulatory requirements and the SOPs established by Bio Farma.

5.6.1.2. Follow-up Visit

The Monitor will carry out regular follow-up visits.
The investigator commits himself to be available for these visits and to allow the monitoring staff direct access to subject medical file and CRFs. The Monitors are committed to professional secrecy.
During the visits, the Monitors:

- will carry out a quality control of the trial progress: respect to protocol and operating guidelines, data collection, signature of consent forms, sample and product management, cold chain monitoring, completion of document and appearance of SAE,
- will check the CRFs and other documents,
- assess the inclusions in order to evaluate the number of complete or ongoing observations.

Monitors will discuss any problem with the investigator and define, after conservation, the actions to be taken. Once the CRFs corresponding to the last visit have been returned duly completed and signed, the investigator must be available to continue the filling out of the correction sheets transmitted by the Monitor, if necessary, until the database is locked.

5.6.1.3. Close-out visit

A close-out visit will be performed at the end of the trial. Its goals are to make sure that:

- the center has all the documents necessary for archiving,
- all samples have been shipped,
- all unused material has been recovered,
- all products have been returned to the sponsor.

5.6.2. Audits and Inspections

If necessary, a quality assurance audit could be carried out by Bio Farma Quality Assurance Department or by independent auditors to make sure that the trial has been conducted according to the protocol and the applicable regulations.An inspection may be conducted by Indonesian Regulatory Authorities.The investigator shall allow direct access to trial documents.

5.6.3. Archiving

The investigator must keep all trial documents provided by Bio Farma for **at least 5 years** after the completion or discontinuation, whatever the center (private, hospital, institution).The investigator will inform Bio Farma of any address change.

6. Adverse Event Reporting

6.1. Definitions

⇒Adverse event (AE):

Any untoward medical occurrence in a patient or clinical investigation subject administered a pharmaceutical product and which does not necessarily have to have a causal relationship with this treatment.

An adverse event can therefore be any unfavorable and unintended sign (including an abnormal laboratory finding, for example), symptom or disease temporally associated with the use of a medicinal product, whether or not considered related to the medicinal product.

⇒Serious adverse event (SAE):

A serious adverse event (experience) is any untoward medical occurrence that at any dose:

- results in death,
- is life-threatening. The term “life-threatening” in the definition of “serious” refers to an event in which the patient was at risk of death at the time of the event; it does not refer to an event, which hypothetically might have cause death, if it were more severe.
- requires inpatient hospitalization or prolongation of existing hospitalization,
- results in persistent or significant disability/incapacity, or
- is a congenital anomaly/birth defect.

Medical and scientific judgment should be exercised in deciding whether expedited reporting is appropriate in other situations, such as important medical events that may not be immediately life-threatening or result in death or hospitalization but may jeopardize the patient or may require intervention to prevent one of the other outcomes listed in the definition above. These should also usually be considered serious.

⇒Adverse drug reaction (ADR):

All noxious and unintended responses to medical product related to any dose should be considered adverse drug reactions. The phrase “responses to a medicinal product” means that a causal relationship between a medicinal product and an adverse event is at least a reasonable possibility, i.e. the relationship cannot be ruled out.

⇒Unexpected adverse drug reaction:
An adverse reaction, the nature or severity of which is not consistent with the applicable product information (e.g. Investigator's Brochure for an unapproved investigational medicinal product).

6.2. Expected Reactions

Anticipated reactions to the trial vaccines are those expected for Influenza HAvaccines and should be mild and transient. Reactions that have been reported may be found in the package insert of Influenza HA vaccine.

6.3. Safety Data Collection and Management Procedures

The safety data will be collected up to one month after the one injection. During this period, each patient will be provided with a diary card to record the appearance, the duration and the intensity (coded 1,2 or 3) of any local reaction and any systemic event expected or not. The intensity of local reactions will be assessed using a plastic bangle presented in Appendix 4.

For any subject, a local reaction is defined as the occurrence of one or several reaction(s) (expected or unexpected) at the injection site within 28 days following vaccination. Local reactions are:

- **Local pain**
- **Redness**
- **Induration**
- **Swelling**
- **Other local reactions**

For any subject, a systemic event is defined as the occurrence of one or several symptom(s) (solicited and unsolicited) within 28 days following vaccination. Systemic events are:

- **Fever** (axillary temperature ≥ 38°C)[18,20]
- **Fatigue**
- **Muscle pain (Myalgia)**
- **Other systemic events**

Parents/subjects will measure the intensity of local reactions in using a plastic bangle (See appendix 4)[18]. The intensity will be coded in the Diary and CRF as follows:

	1=Mild	2=Moderate	3=Severe
**Local pain for infants and children** (6-35 months)	Reacts when site is touched	Cries when site is touched	Cries when limb is moved
**Local pain for subjects ≥ 3 years of age**	Mild pain to touch	Pain with movements	Significant paint at rest
**Redness/swelling/ Induration Other local reactions**	Reaction completely included in the smaller circle	Largest diameter of the reaction included between the two circles	Reaction beyond the largest circle
**Fever**	38.0 – 38.4°C	38.5 – 38.9°C	> 39.0°C
**Fatigue Myalgia**	No interference with activity	Some interference with activity	Siginificant prevents daily activity
**Other systemic events**	No interference with activity	Some interference with activity not requiring medical intervention	Prevents daily activity, requires medical intervention

Collection and follow-up of adverse events
Adverse events will be collected as indicated in the CRF. Adverse events likely to be related to the product, whether serious or not, which persist at the end of the trial will be followed up by the investigator until their complete disappearance. The investigator will inform the Medical Responsible or the Monitor of the date of final disappearance of the adverse event and will document it on a correction sheet.

Moreover, any serious adverse event likely to be related to the product and occurring after trial termination should be reported by the investigator to Bio Farma according to the procedure described below.

6.4. Reporting of Serious Adverse Events

Every serious adverse event occurring throughout the trial should be reported to the Ethic Committee, Regional Vaccine safety Advisory Committee and to the sponsor, by the investigator as **soon as he/she is alerted of it** i.e. **within 24 hours**, even if the investigator considers that the adverse event is not related to treatment.
Notification should be made:

- by phone/email/ by fax; then the investigator should immediately send the **completed alert form** to Ethic Committee, Regional Vaccine Safety Advisory Committee and to the sponsor.

The copy of alert form should be sent to Bio Farma. The investigator should then fill in the **SAE reporting form** as soon as possible, i.e. within five working days or seven calendar days. This form should be signed by the investigator and sent by fax or express mail to the sponsor, Ethics Committee and Regional Vaccine Safety Advisory Committee. Bio Farma as a sponsor should send the reporting form for SAEs-related product (Adverse Drug Reaction) to the NRA within 15 calendar days since the cases was found.

6.5 Causality Assessment

Causality is the relationship between two events (the cause and the effect), where the second event is a consequence of the first. A direct cause is a factor in absence of which the effect would not occur (necessary cause). Sometimes there are multiple factors that may precipitate the effect (event) or may function as co-factors so that the effect (event) occurs).

Causality assessment is the systematic review of data about AEFI case; it aims to determine the likelihood of a casual association between the event and the vaccines(s) received. For individual cases, one tries to apply the evidence available on the basis of the history and time frame of the event to arrive at a casual likelihood.

Case selection of cases for causality assessment should focus on:

- Serious AEFI
- The occurrence of events above the expected rate or of unusual severity;
- Signals generated as a result of individual or clustered cases as these could signify a potential for large public health impact.

WHO Classification[21]:

I. Case with adequate information for causality conclusion

A. Consistent casual association to immunization: A case with adequate information for causality conclusion

A1. Vaccine product-related: An AEFI that is caused or precipitated by a vaccine due to one or more of the inherent properties of the vaccine product

A2. Vaccine quality defect-related reactions: An AEFI that is caused or precipitated by a vaccine due to one or more quality defects of the vaccine product, including the administration device, as provided by the manufacturer.

A3. Immunization error-related reaction: An AEFI that is caused by inappropriate vaccine handling, prescribing or administration and that thus, by its nature is preventable.

A4. Immunization anxiety-related reaction: An AEFI arising from anxiety about the immunization.

B. Indeterminate

B1. Temporal relationship is consistent but there is insufficient definitive evidence that vaccine caused the event (it may be a new vaccine- linked event). This a potential signal and needs to be considered for further investigation.

B2. Reviewing factors result in conflicting trends of consistency and inconsistency with casual association to immunization (i.e. it may be vaccine-associated as well as coincidental and it is not possible clearly to favour one or the other.

C. Inconsistent causal association to immunization (coincidental): This could be due to underlying or emerging condition(s) or conditions caused by exposure to something other than vaccine.

[21] WHO. Causality Assessment of an Adverse Event Following Immunization. Geneva: WHO; 2013.

Case without adequate information for causality conclusion

This case is categorized as “unclassifiable” and requires additional information for further review of causality. The available information on unclassifiable cases should be placed in a repository or an electronic database which should be periodically reviewed to see if additional information is availavle for classification and to perform analyses for identifying signals.

The decision to modify or discontinue the trial, or to break individual or all study codes may be made after mutual agreement between the sponsor and the investigator(s).

Regulatory Requirements

In order to comply with current regulations on serious adverse event reporting to Health Authorities and to allow Bio Farma to carry out a precise analysis of the safety of the developed products, the investigator pledges to document accurately the event, to respect notification deadlines, to provide Bio Farma with all necessary information and if requested by the sponsor, to give access to source documents.

Bio Farma pledges to inform Health Authorities as soon as it is informed of any serious adverse event likely to be related to the product. The sponsor also pledges to inform the Authorities of any trial discontinuation and specify the reason for discontinuation.

Blood sampling

A blood sample to be taken as soon as possible might be requested in case of serious adverse event if it can help in analyzing the SAE. Four milliliters of blood will be taken in a dry tube.

Determination of SAE

Determination of SAE and its causal relationship with the product (vaccine) will be held by Regional Advisory Committee on Adverse Event. This special committee member consists of experts from multidisciplinary field, i.e. medicine (pediatrics, immunology, neurology, vaccine experts, forensic etc.), public health, biostatistics, health law, etc.

The procedural report of SAE based on the regulation in accordance with Article 21 and 22 Head of National Food and Drugs Agency (Indonesian FDA) Decree No. 21/2015.

7. Evaluation Criteria

7.1. Primary Evaluation Criteria

7.1.1. Definition of the Criteria

The main evaluation criteria is defined as the percentage of subjects with an anti-influenza titer ≥1:40 HI units, 28 days after the last dose immunization.

7.1.2. Parameters to be measured

The anti-influenza antibody serological responses will enable to assess whether the vaccine induce protective antibody levels.

7.1.3. Method and Timing of Measurement

Antibody titers are measured at visit V1 (Day 0), Visit V2 (V1+28(-4/+7) days) for group I-III and at visit V3 (V2+28 days -4 /+7 days) for group IV & V.
The anti-influenza antibody serological responses will be tested by Hemagglutination Inhibition method and will be expressed as HI units.

7.2. Secondary Evaluation Criteria

7.2.1. Definition of the Criteria

<u>**Immunogenicity**</u>

a. Serological response to influenza HA vaccine: GMT, percentage of subjects with increasing antibody titer ≥ 4 times and or percentage of subjects with transition of seronegative to seropositive 28 days after the last dose of Influenza HA vaccine.
b. Description of serological response between quadrivalent and trivalent influenza HA vaccine in subjects 9-40 years old.
c. Description of serological response between each batch number of Quadrivalent Influenza HA vaccine in subjects 9 -40 years old.

<u>**Safety**</u>

Safety criteria are defined as the percentage of subjects with local reaction and/or systemic events after vaccination:

a. Number and percentage of subjects with at least one immediate reaction (local reaction or systemic event) within 30 minutes after each vaccination.
b. Number and percentage of subjects with at least one of these adverse events, expected or not, within 72 hours after vaccination.

c. Number and percentage of subjects with at least one of these adverse events, expected or not, between 72 hours to 28 days after vaccination.
d. Any serious adverse event occurring from inclusion until 28 days after vaccination.
e. Description of adverse events between quadrivalent and trivalent influenza HA vaccine.
f. Description of adverse events between each batch numberof quadrivalent influenza HA vaccine.

7.2.2. Parameters to be measured

a. GMT, seroprotection and seroconversion rates before and after one dose of immunization will be described per antigen per group vaccine with 95% confidence interval (CI).

b.1 Local reactions and systemic events occurring within 30 minutes after vaccination, 72 hours after vaccination and unxpected event rates, which occurred after 72 hours will be assessed per antigen and per group vaccine with 95% CI.
Any Serious Adverse Events occurring over the study period will be described.

Subjects will record the presence of any local or systemic symptom within 28 days following each injection in the diary card. They will answer all questions in the diary for expected and unexpected events during this period.

The investigator will report the following data in the CRF:
- The start and end dates of all events.
- The severity (code 1, 2 or 3) of all local reactions.
- Site of local reactions.
- The severity (code 1, 2 or 3) of all systemic events.
- The exact daily maximal axillary temperature up to 3 days after the vaccination.
- The exact maximal axillary temperature of any other fever period.
- The action taken.

b.2 Safety assessment:
The investigator will assess the intensity (code 1, 2 or 3), duration and relation.
- Percentage of subjects with at least one immediate reaction (local reaction or systemic event) within 30 minutes after each vaccination.
- Local and systemic reactions, expected or not, occurring within 72 hours after each injection will be evaluated by interviewing the subject/parents during the post surveillance visits: V0, V2 and V3. Particularly, the axillary temperature will be measured for three days after vaccination, in the evening and/or at time of febrile peak, and the highest temperature will be recorded in the diary

card, expressed as Celsius degrees, using a thermometer. The trial team will record the information in the CRF.

- Any serious adverse event occurring during the study.

8.1. Statistical Methods and Data Analysis

8.1.1. Determination of Sample Size

8.1.1.1. Proportion Formula

Sample size is determined based on 95% confidence interval and power of the test 80%. Using sample size formula for comparing two a population proportion

$$n = \frac{\left(Z_{1-\alpha/2}\sqrt{2\bar{P}(1-\bar{P})} + Z_{1-\beta}\sqrt{p1(1-p1)+p2(1-p2)}\right)^2}{(p1-p2)^2}$$

with p1= immunogenicity of Trivalent Influenza HA vaccine = 96% and the maximum difference between two proportion equal 15% (or p2= immunogenicity of QIV = 81%), from the formula, the required sample size would be 115 in each group including 20 % of drop out anticipation

8.1.1.2. To evaluate immunogenicity and safety in three consecutive batches of quadrivalent Influenza HA vaccine in group 9-40 years of age. Sample size is determined based on formula for studies one proportion :

$$n = \frac{\left(Z_{1-\alpha/2}\sqrt{p_0(1-p_0)} + Z_{1-\beta}\sqrt{p_1(1-p_1)}\right)^2}{(p_1 - p_0)^2}$$

With p_0 = immunogenicity in each batches of quadrivalent = 0.96; $(p_1 - p_0)$ = effect size, is determined at least 0.06 (or p_1 equal 0.90). For 95% confident interval and power of the test 80% ($Z_{1-\alpha/2} = 1.96$; $Z_{1-\beta} = 0.84$), from the formula above, the estimated sample size is n = 112 for each batches.

With the assumption that not all of the subject could complete the study, the total number of subject will be added at least 20%, (N x 1.2) = 134 from the minimum requirement. Approximately 135 subjects per group will be involved in this study.

Since using the second formula need more subjects, then for this study 135 subjects will be involved for each group, and totally 810 subjects for the whole study, with expectation the sample size will be suitable for both formulas.

8.1.2. Data sets to be analyzed

8.1.2.1. Definition of the population

“Full Analysis Set” (Intention To Treat, ITT): Every subject included in the study will be analyzed in this population, except if he/she did not receive any injection of one of the study vaccines.

“Per Protocol Subjects” (PP): Following non compliant subjects will be excluded from this population:

- Subjects included without meeting at least one inclusion criterion
- Subjects included despite meeting at least one non inclusion criterion
- Subjects found non compliant with the immunization or blood sampling schedule.
- Subjects vaccinated at least once with the wrong vaccine (non compliance with the randomization schedule).
- Subjects excluded from the ITT analysis.

8.1.2.2. Populations used in the analysis

Safety Analysis:
All included and vaccinated subjects will be analyzed, each subject being analyzed according to the vaccine effectively received.

Immunogenicity Analysis:
The primary immunogenicity analysis will be conducted on the Per Protocol population.The secondary immunogenicity analysis will be conducted on the All Randomized population, each subject being analyzed according to the group attributed by the randomization process.

8.1.3. Statistical Methodology

8.1.3.1. Primary Criterion

The main objective of the study is the protectivity rate of the Influenza HA vaccine. Percentage of subjects with anti Influenza titer ≥ 1:40 HI units 28 days after last injection.

8.1.3.2. Secondary Criteria

Immunogenicity analysis

- ♦ Seroprotection and seroconversion rates at last visit (V2 or V3): the following parameters will be presented: crude rates with their 95% confidence intervals (computed using the exact binomial probability)
- ♦ Geometric means of titers (GMTs) with their 95% CI will be presented at V0 and V2 or V3.

♦ Description between groups.

Safety analysis
At each follow-up visit, the following parameters will be computed:

- ♦ Percentage and number of subjects experiencing immediate reactions within 30 minutes of vaccinations.
- ♦ Number and percentage of subjects with at least one local reaction (within 72 hours after each injection), with the frequencies of each type of local reactions (global and broken down according to the size of the reaction when appropriate)
- ♦ Number and percentage of subjects with at least one systemic event (within 72 hours after each injection either related or non related to the vaccine), with the frequencies of each type of event (global and broken down according the relationship and the severity)
- ♦ Number and percentage of subjects with at least one local reaction (within 72 hours to one month after each injection), with the frequencies of each type of local reactions (global and broken down according to the size of the reaction when appropriate)
- ♦ Number and percentage of subjects with at least one systemic event (within 72 hours to 1 month after each injection either related or non related to the vaccine), with the frequencies of each type of event (global and broken down according to the relationship and the severity)
- ♦ Number and percentage of subjects with at least one Serious Adverse Event, with the frequencies of each type of event (global and broken down according to the relationship and the severity of the event).
- ♦ Description between groups.

8.2. Data Management

Throughout regular data collection and monitoring, clinical data reported on CRFs and/or relevant serological/biological samples analysis results scheduled in the protocol will be integrated into a clinical data management system.

For each batch of data, single entry, quality control and triggers to computerized logic and/or consistency checks will be systematically applied in order to detect errors or omissions. After integration of all corrections in the complete set of data, the data based will be locked and saved before being released for statistical analysis.

Each step of this process will be monitored through the implementation of individual passwords and/or regular backups in order to maintain appropriate database access and to guarantee database integrity.

9. Confidentiality, Stipends and Adverse Events Compensation and Insurance

9.1. Confidentiality

9.1.1. Confidentiality of Data

Prior to initiation of the trial, the investigator will sign a fully executed confidentiality agreement with Bio Farma.

9.1.2. Confidentiality of Patient Records

Confidentiality of patient records will be ensured by identifying documents using inclusion number and subject initials (first three letters of the subject's name).

9.2. Stipends for Participation

Not applicable.

9.3. Adverse Events Compensation and Insurance

Bio Farma shall insure all clinical trial subjects and investigators and cover medicinal treatment expenses and/or compensation to clinical trial subject to anticipate any serious adverse events or adverse events as consequence of their participation on this clinical study. Bio Farma shall not be liable for all claims from third parties caused by or resulting from malpractice and/or negligence or willful misconduct of investigators and its staff.

If there is any doubt raised by cause and effect relation between adverse reactions and their participation on this clinical study, judgment of Indonesian Vaccine Safety Advisory Committee or NRA shall be needed.

10. Publication Policy

The final report will be prepared by a publication committee which includes the investigators and representatives of Bio Farma. It will be signed by the coordinating (or principal) investigator. Publication will not reasonably withhold.

11. Bibliographical References

1. World Health Organization. Global action plan for influenza vaccines. Geneva: World Health Organization; 2016.
2. Ortiz JR, Perut M, Dumolard L, et al. A global review of national influenza immunization policies: Analysis of the 2014 WHO/UNICEF Joint Reporting Form on immunization. Vaccine. 2016; 34:5400-05. http://dx.doi.org/10.1016/j.vaccine.2016.07.045
3. World Health Organization. Influenza virus infections in humans (February 2014). Geneva: World Health Organization; 2014.
4. World Health Organization. Questions and Answers Recommended composition of influenza virus vaccines for use in the northern hemisphere 2016-2017 influenza season and development of candidate vaccine viruses for pandemic preparedness. Geneva: World Health Organization; 2016.
5. Pretty Multiharina et al, Laboratory Network in ILI and SARI Surveillance System for Influenza Virus Evolution Monitoring, IIF Symposium 2013.
6. Pink Book, Chapter: Influenza, Center for Disease Control. www.cdc.gov/vaccines/pubs/pinkbook/index.html, downloaded July 2016.
7. WHO, Weekly epidemiological record, Vaccines against influenza WHO position paper-November 2012, Geneva 2012, 47(87); 461-476.
8. Fukuda K, Levandowski RA, Bridges CB et al. Inactivated influenza vaccines. In Plotkin SA, Orenstein WA, with assistance of Offit PA. vaccines, Saunders: Philadelphia, 2004: 339-700
9. World Health Organization. A Manual for Estimating Disease Burden Associated with Seasonal Influenza. Geneva: World Health Organization; 2015.
10. World Health Organization. Influenza Update – 273. Geneva: World Health Organization; 2016 [cited 2016 October 12]. Available from: http://www.who.int/influenza/surveillance_monitoring/updates/latest_update_GIP_surveillance/en/
11. Nair H, et al. Global burden of respiratory infections due to seasonal influenza in young children; a systemic review and meta analysis. The Lancet 2011, 378:1917-30.

12. World Health Organization. Vaccines. Geneva: World Health Organization [cited 2016 October 12]. Available from: http://www.who.int/influenza/vaccines/en/
13. Center for Disease Control and Prevention. Quadrivalent Influenza Vaccine. Atlanta: Center for Disease Control and Prevention; 2016 [cited 2016 October 12]. Available from: http://www.cdc.gov/flu/protect/vaccine/quadrivalent.htm
14. Dhamayanti M, Rusmil K, Idjradinata P. Respon Imun terhadap Vaksin Influenza pada Remaja. Jurnal Kedokteran Brawijaya 2012, 27(2):102-6.
15. Bachtiar NS, Sukriyani Y, Lestari, Sari RM. Validation of Hemagglutination Inhibition Assay for influenza antibody titer. Poster presentation at Indonesian Influenza Foundation Symposium, 2013.
16. Fadlyana E, Rusmil K, Bachtiar NS, Gunadi R, Sukandar H. Immunogenicity and safety of a trivalent inactivated influenza vaccine. Paediatrica Indonesiana 2011;51(1):22-8.
17. Fadlyana E, Rusmil K, Bachtiar NS, Gunadi R, Sukandar H. Immunogenicity and safety of a trivalent inactivated influenza vaccine. Poster Presentation Indonesian Influenza Foudation Symposium 2013.
18. Irie S, Fujieda M, Ito K, Ishibashi M, Takamizawa T, Ishikawa T,et al. Immune response against inactivated influenza vaccine in children under four years old. The Journal of Infectious Disease, 2007:81;284-90.
19. Medise BE, Gunardi H, Sekartini R, Soedjatmiko, Satari HI. Immunogenicity and Safety of Flubio (Influenza HA) Vaccine in Infants and Children (Bridging Study). Department of Child Health, School of Medicine, University of Indonesia. 2015 (not published)
20. The Research Foundation for Microbial Diseases of Osaka University. Clinical Trial for BK-FLU on Healthy Adults. (not published)
21. Bachtiar NS, Sukriyani Y, Lestari, Sari RM. Validation of Hemagglutination Inhibition Assay for Influenza Antibody Titer. Poster presentation at Indonesian Influenza Foundation Symposium 2013.
22. FDA, Guidance for Industry, Toxicity Grading Scale for Healthy adult and adolescent volunteers enrilled in Preventive Vaccine Trials, April 2005.

23. WHO, Department of Vaccines and Biologicals, Supplementary information on vaccine safety, Part2:Background rates od adverse events following immunization, Geneva 2000, pp 46-50.
24. WHO. Causality Assessment of an Adverse Event Following Immunization. Geneva: WHO; 2013.

12. Appendices

The following is a non-comprehensive list of possible appendices

Appendix 1: Personnel involved in the Trial
Appendix 2: SAE reporting form
Appendix 3: Plastic bangle

Appendix 1: Personnel Involved in the Trial

**Principal Investigator**	Dr. Meita Dhamayanti, dr.,Sp.A(K).,MKes.
**Medical Advisor**	Prof. Cissy B. Kartasasmita, dr.,Sp.A(K).,MSc.,PhD.
**Subinvestigators**	1. Prof. Dr. Kusnandi Rusmil, dr.,Sp.A(K).,MM.<br>2. Dr. Eddy Fadlyana, dr.,Sp.A(K).,MKes.<br>3. Rodman Tarigan, dr.,Sp.A(K).,Mkes.<br>4. Susantina Prodjosoewojo, dr.,Sp.PD<br>5. Andri Reza Rahmadi, dr.,Sp.PD.,MKes.
**Biometry**	Dr. Hadyana Sukandar, MSc.
**Biological Laboratory**	Rini Mulia Sari, dr.<br>Yani Sukriyani<br>Restika Andiarini
**Monitor**	Dr.Novilia Sjafri Bachtiar, dr., MKes.<br>Rini Mulia Sari, dr.<br>Julianita Fahmi, dr.<br>Asep Irham, F. Q., dr

Appendix 2: Sample SAE reporting forms

FORMULIR PELAPORAN KEJADIAN IKUTAN PASCA IMUNISASI (KIPI)

Kode sumber data : ……………..
Tgl.terima : ……………………..

PASIEN

Nama : ……………………………
Nama Orangtua : …………………….............
Alamat : ……………………………
……………………………
……………….....…………………..

Tanggal lahir: ……./……../………
Jenis kelamin:.
Laki-laki ....
Perempuan ....
Bagi Wanita Usia Subur (WUS):

Penanggung jawab (dokter)
Alamat (RS, Puskesmas, Klinik)
....
....

Kota : …………………………................
RT/RW : ……...Kel……………….................
Prop. : ………………………………............
Kota : ………..Kode Pos……..................
Telp. : ………………………………............

Hamil ……………
Tidak hamil ……..
Tidak tahu ………

Prop. : ………………………......
Telp. : ………………………..

Pemberi imunisasi: Dokter/bidan/perawat/jurim

Daftar vaksin yang pernah diberikan dalam 4 minggu terakhir, termasuk imunisasi terakhir

	Jenis vaksin	Pabrik	No.Lot/Batch	Pemberian			Tempat pemberian imunisasi (*)
				Cara tetes oral/i.m/s.c/i.c	Jumlah dosis	Tanggal imunisasi	
1							
2							
3							
4							

(*) Tempat pemberian imunisasi di:
1. Dokter Praktek/RS 2. Puskesmas 3. Posyandu 4. Balai Pengobatan/Bidan 5. Balai Imunisasi 6. Sekolah 7. Dll (sebutkan)

Manifestasi kejadian ikutan (keluhan, gejala klinis)

		Waktu Mulai	Lamanya: Mm	Jam	Hr
☐	Kesadaran menurun				
☐	Kejang-kejang				
☐	Demam tinggi lebih sehari				
☐	Adanya kelumpuhan atau kelemahan otot				
☐	Infeksi berat ditempat injeksi				
☐	Infeksi berat diluar tempat injeksi				
☐	Reaksi alergi:				
☐	- pembengkakan bibir & tenggorokan				
☐	- sesak nafas				
☐	- erythema				
☐	- papula				
☐	- tekanan darah menurun				
☐	Diare				
☐	Muntah				
☐	Encephalopathy (*)				
☐	Meningitis dan atau encephalitis (*)				
☐	Kelumpuhan (*)				
☐	Trombositopenia (*)				
☐	Lain-lain: ………………………………..				

Keterangan lanjutan hasil akhir:

- Pasien meninggal tgl.: …../…./…..
- Sakit
- Perlu tindakan darurat/dokter
- Perlu perawatan RS (…..hari)
- Sembuh
- Belum sembuh
- Tidak diketahui
- Lain-lain …………………………
…………………………

(*) Ditentukan berdasarkan diagnosa dokter

Pengobatan yang diberikan:<br>▫ …………………………………………... ▫ ………………………………………… ▫ …………………………………………	
Riwayat efek samping obat/vaksin yang pernah dialami:	
Obat-obatan yang diberikan bersamaan:<br>▫………………….. ▫………………… ▫ ……………………	Data laboratorium (bila ada)
Penyakit yang diduga diderita pada saat vaksinasi (spesifik)	Diagnosa dokter tentang:<br>Alergi, kelainan sejak lahir, pengobatan khusus (spesifik): ada/tidak<br>Bila ada sebutkan: ……………………………………………

Waktu penerimaan laporan Kejadian Ikutan<br>Tanggal: ………./………../…………	…………………………., tgl. ………/………./…………<br>Tanda Tangan Pelapor<br><br><br>( )

FORMULIR LAPORAN KIPI SERIUS

Dikirim dengan segera (<24 jam) kepada
Judul Uji Klinis : Dr. Meita Dhamayanti, dr., SpA(K)., MKes.

Protectivity and Safety of Quadrivalent Influenza HA Vaccine in Indonesian Population (Bridging Study)

Kode Uji Klinis : QIV 0217

DATA SUBYEK

Inisial Nama	No.Inklusi	Tanggal lahir	Jenis kelamin
☐☐☐	☐ / ☐☐☐	☐☐ ☐☐ ☐☐☐☐	☐ La i-laki<br>☐ Perempuan

Data Kejadian

Tanggal timbulnya gejala berat : ☐☐ ☐☐ ☐☐☐☐

Tanggal berakhirnya gejala berat : ☐☐ ☐☐ ☐☐☐☐

Atau berlanjut : ☐

Gambaran gejala : ……………………………………….

(tipe, intensitas, kronologis) ……………………………………..

……………………………………..

……………………………………..

Sampel darah (untuk diagnosa): ada ☐ tidak ☐

Isi dengan tanda √ pada kotak sesuai dengan Kejadian:

☐ Meninggal, bila ya tanggal: ☐☐ ☐☐ ☐☐☐☐

☐ Mengancam nyawa

☐ Perawatan rumah sakit

☐ Gejala menetap

☐ Cacat bawaan/lahir

☐ Lain-lain

(tuliskan):………………………

…………………………………………..

Hasil akhir: Sembuh ya ☐ tidak ☐

DATA PRODUK UJI KLINIK

Nama produk :…... Nomor Batch: ☐☐☐☐☐☐☐☐

Nama produk :…... Nomor Batch: ☐☐☐☐☐☐☐

Tanggal imunisasi terakhir: ☐☐ ☐☐ ☐☐☐☐

Tempat pemberian imunisasi:

1. Dokter praktek/RS
2. Puskesmas
3Posyandu
4Balai Pengobatan/Bidan

5. Balai Imunisasi
6. Sekolah
7.dll. Sebutkan:

Cara pemberian :………………………………

Sisi penyuntikan : Kiri ☐ Kanan ☐ ☐

Berhubungan dengan produk vaksin Ya Tidak Tidak diketahui

NAMA DAN ALAMAT PENELITI Tanggal pelaporan : ☐☐ ☐☐ ☐☐☐☐

Telepon : Tanda tangan :

Fax. :

* : Coret yang tidak perlu

Appendix 3: Plastic bangle model

Group II (13-17 years old) and
Group III (18-40 years old)

This bangle will be printed on a transparent plastic square and will be used to assess the intensity of local reactions, which may occur after the injection of vaccine. The bangle is composed of one 10 cm diameter circle and one 5 cm diameter circle. These two circles are concentric and the cross corresponds to the center of the circles.

This plastic bangle will be given to the health worker with instruction for use, as follows :

1. Place the cross at the center of the reaction (middle of the largest diameter, see diagram)
2. Do not press on the plastic square so as not to increase the diameter of the reaction.
3. Note the intensity (1, 2 or 3).

Intensity 1 and 2 are represented by the circle that completely encircles the largest diameter of the reaction. If the largest diameter of the reaction goes outside the biggest circle, the intensity is 3.

For example, in this figure, the intensity is coded 2.

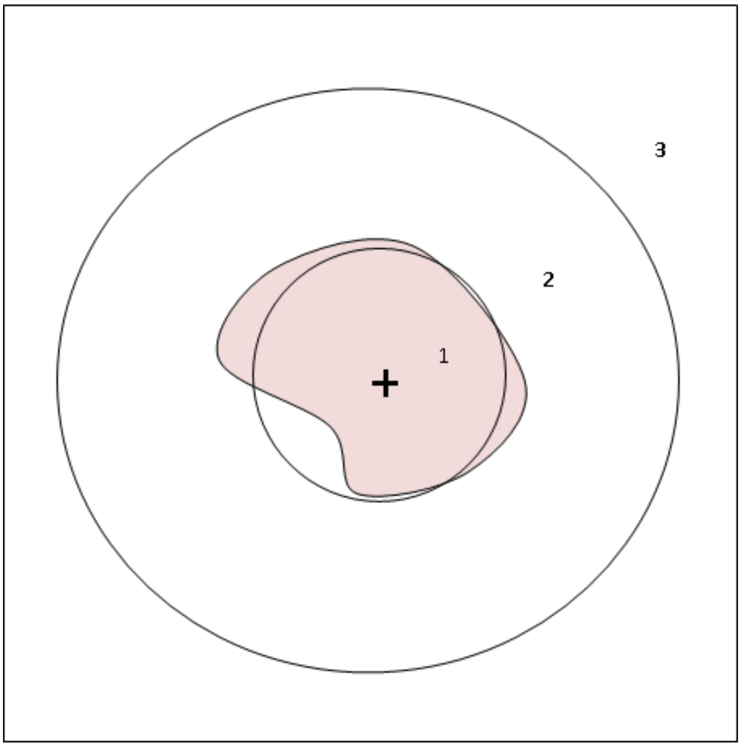

Plastic Bangle (for infants and children)

Group I (9-12 years)
Group IV (6-35 months)
Group V (3-8 years)

This bangle will be printed on a transparent plastic square and will be used to assess the intensity of local reactions, which may occur after the injection of vaccine. The bangle is composed of one 5 cm diameter circle and one 2.5 cm diameter circle. These two circles are concentric and the cross corresponds to the center of the circles.

This plastic bangle will be given to the health worker with instruction for use, as follows:

1. Place the cross at the center of the reaction (middle of the largest diameter, see diagram)
2. Do not press on the plastic square so as not to increase the diameter of the reaction.
3. Note the intensity (1, 2 or 3).

Intensity 1 and 2 are represented by the circle that completely encircles the largest diameter of the reaction. If the largest diameter of the reaction goes outside the biggest circle, the intensity is 3.

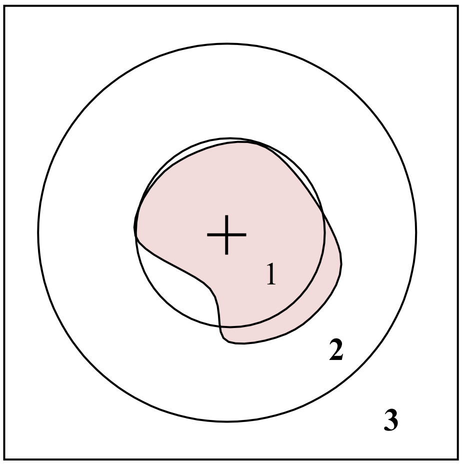

For example, in this figure, the intensity is coded 2.
