# Supplementary material for: Immunogenicity and safety of Quadrivalent Influenza HA vaccine compared with Trivalent Influenza HA vaccine and evaluation of Quadrivalent Influenza HA vaccine batch-to-batch consistency in Indonesian children and adults
Source: PLoS One. 2023 Aug 24;18(8):e0281566. doi: 10.1371/journal.pone.0281566 (PMC10449119; doi:10.1371/journal.pone.0281566)
Supplement: Attachment — Submitted filename: Response ro Reviewers.docx [file pone.0281566.s004.docx]

**PONE-D-22-35728R1**

Immunogenicity and safety of Quadrivalent Influenza HA vaccine compared with Trivalent Influenza HA vaccine and evaluation of Quadrivalent Influenza HA vaccine batch-to-batch consistency in Indonesian children and adults

PLOS ONE

Dear editorial staff and the reviewers,

We appreciate the editorial staff and the reviewers for taking time to carefully review the manuscript and give detailed and constructive comments, which has greatly helped to improve this paper. Below is our point-by-point response to each comment.

**Journal Requirements:**

Please review your reference list to ensure that it is complete and correct. If you have cited papers that have been retracted, please include the rationale for doing so in the manuscript text, or remove these references and replace them with relevant current references. Any changes to the reference list should be mentioned in the rebuttal letter that accompanies your revised manuscript. If you need to cite a retracted article, indicate the article’s retracted status in the References list and also include a citation and full reference for the retraction notice.

[Note: HTML markup is below. Please do not edit.]

**Response :** Thank you for reminding us. We have reviewed our reference list, and we have changed some cite and references which couldn’t be accessed anymore to the new one.

**Reference no. 7**

World Health Ogranization. Recommended composition of influenza virus vaccines for use in the 2013-2014 northern hemisphere influenza season. February 2013. Available from: https://www.who.int/publications/m/item/recommended-composition-of-influenza-virus-vaccines-for-use-in-the-2013-2014-northern-hemisphere-influenza-season (accessed June 12, 2023).

**Reference no. 13**

Luo FJ, Yang LQ, Ai X, Bai YH, Wu J, Li SM, et al. Immunogenicity and safety of three 2010-2011 seasonal trivalent influenza vaccines in Chinese toddlers, children and older adults. [Hum Vaccin Immunother.](https://www.ncbi.nlm.nih.gov/pmc/articles/PMC3906273/) 2013 Aug 1; 9(8): 1725–1734. doi: 10.4161/hv.24832.

**Reference no. 16**

Food and Drug Administration Center. Clinical Data Needed to Support the Licensure of Seasonal Inactivated Influenza Vaccines. Guidance for Industry. 2007: 1-16. Available from: <https://www.fda.gov/regulatory-information/search-fda-guidance-documents/clinical-data-needed-support-licensure-seasonal-inactivated-influenza-vaccines>. (accessed June 12, 2023)

**Comments to the Author**

1. If the authors have adequately addressed your comments raised in a previous round of review and you feel that this manuscript is now acceptable for publication, you may indicate that here to bypass the “Comments to the Author” section, enter your conflict of interest statement in the “Confidential to Editor” section, and submit your "Accept" recommendation.

**Response :** Thank you for the comment. We have adequately addressed our comments raised in the previous round of review.

2. Is the manuscript technically sound, and do the data support the conclusions?

The manuscript must describe a technically sound piece of scientific research with data that supports the conclusions. Experiments must have been conducted rigorously, with appropriate controls, replication, and sample sizes. The conclusions must be drawn appropriately based on the data presented.

**Response :** Yes, our manuscript has already described a technically sound and the data has already supported the conclusions

3. Has the statistical analysis been performed appropriately and rigorously?

**Response :** Yes, the statistical analysis has been already performed appropriately and rigorously.

4. Have the authors made all data underlying the findings in their manuscript fully available?

The [PLOS Data policy](http://www.plosone.org/static/policies.action#sharing) requires authors to make all data underlying the findings described in their manuscript fully available without restriction, with rare exception (please refer to the Data Availability Statement in the manuscript PDF file). The data should be provided as part of the manuscript or its supporting information, or deposited to a public repository. For example, in addition to summary statistics, the data points behind means, medians and variance measures should be available. If there are restrictions on publicly sharing data—e.g. participant privacy or use of data from a third party—those must be specified.

**Response :** Thank you for reminding us. We have added one table (Table 3. Geometric Mean Titers (GMT) to influenza A (H1N1), A (H3N2), and B virus strains Pre- and 28 days post-immunization) to explain the result in page 9, line 187-192.

**Revised manuscript (Page 9, line 185-190)**

Comparison of GMT between subjects who received QIV and TIV was shown on table 3 and figure 2. No significant differences in GMT 28 days after immunization between one dose of QIV and TIV in the 9–40-year-old subjects for A/California/7/2009 (X-179A) (H1N1) pdm09, A/Hong Kong/4801/2014 (X-263) (H3N2), and B/Texas/2/2013 (*p* = 0.322, *p* = 0.536, and *p* = 0.378, respectively). However, in strain B/Phuket/3073/2013, GMT 28 days after immunization was significantly higher in QIV group than in TIV group (*p* < 0.001).

**Table 3. Geometric Mean Titers (GMT) to influenza A (H1N1), A (H3N2), and B virus strains Pre- and 28 days post-immunization**

| **Strain** | **Pre-immunization** | | P^*)^ | **Post-immunization** | | P^*)^ |
| --- | --- | --- | --- | --- | --- | --- |
|  | QIV | TIV |  | QIV | TIV |  |
| A/California/7/2009/H1N1  GMT/95%CI | 57.12  (51.04-63.93) | 49.35  (41.25-59.05) | 0.250 | 910.75  (833.68-944.72) | 939.07  (777.68-1133.7) | 0.322 |
| A/Hongkong/4801/2014/H3N2  GMT/95%CI | 51.67  (46.75-57.10) | 52.95  (44.67-62.78) | 0.701 | 502.81  (453.42-557.44) | 554.75  (467.52-658.11) | 0.536 |
| B/Texas/2/2013  GMT/95%CI | 26.34  (24.17-28.71) | 25.47  (22.20-29.21) | 0.853 | 330.37  (292.15-373.51) | 358.26  (281.77-455.41) | 0.378 |
| B/ Phuket/3073/2013  GMT/95%CI | 42.87  (39.32-46.75) | 42.54  (36.06-50.19) | 0.642 | 348.42  (317.76-382.12) | 112.85  (94.65-134.56) | <0.001 |

**Fig 2. Hemagglutination inhibition (HI) Geometric Mean Titers (GMT) before and 28 days after QIV and TIV immunization.**

5. Is the manuscript presented in an intelligible fashion and written in standard English?

PLOS ONE does not copyedit accepted manuscripts, so the language in submitted articles must be clear, correct, and unambiguous. Any typographical or grammatical errors should be corrected at revision, so please note any specific errors here.

**Response :** We have already made sure that language we used in this manuscript have been clear, correct and unambiguous.

6. Review Comments to the Author

Please use the space provided to explain your answers to the questions above. You may also include additional comments for the author, including concerns about dual publication, research ethics, or publication ethics. (Please upload your review as an attachment if it exceeds 20,000 characters)

Reviewer #1: Line 149-150, “The 149 differences of antibody concentration for each antigen before and after primary series of Influenza vaccine was analysed using Wilcoxon test”. Please indicate clearly which Wilcoxon test was used.

**Response :** In this manuscript, there is no data which used Wilcoxon test to analyse the data. We have deleted this part. **(Page 7, line 146-151)**

Line 150-151, “Values of p < 0.05 indicated statistically significant differences between groups were assessed by Chi-square test”. This sentence is not clear. Why was this cut-off p-value only used for analysis assessed by Chi-square test? You may want to say “Values of p < 0.05 indicated statistically significant differences between groups.”

**Response :** Thank you for the suggestion. Yes, we have revised this line as suggested. **(Page 7, line 149)**

7. PLOS authors have the option to publish the peer review history of their article ([what does this mean?](https://journals.plos.org/plosone/s/editorial-and-peer-review-process#loc-peer-review-history)). If published, this will include your full peer review and any attached files.


If you choose “no”, your identity will remain anonymous but your review may still be made public.


**Do you want your identity to be public for this peer review?** For information about this choice, including consent withdrawal, please see our [Privacy Policy](https://www.plos.org/privacy-policy).

Reviewer #1: No

[NOTE: If reviewer comments were submitted as an attachment file, they will be attached to this email and accessible via the submission site. Please log into your account, locate the manuscript record, and check for the action link "View Attachments". If this link does not appear, there are no attachment files.]

While revising your submission, please upload your figure files to the Preflight Analysis and Conversion Engine (PACE) digital diagnostic tool, <https://pacev2.apexcovantage.com/>. PACE helps ensure that figures meet PLOS requirements. To use PACE, you must first register as a user. Registration is free. Then, login and navigate to the UPLOAD tab, where you will find detailed instructions on how to use the tool. If you encounter any issues or have any questions when using PACE, please email PLOS at [figures@plos.org](mailto:figures@plos.org). Please note that Supporting Information files do not need this step.
